# Impact of low environmental temperatures on the global burden of myocarditis: insights from the 1990–2021 global burden of disease study

**DOI:** 10.1186/s12872-025-05209-2

**Published:** 2025-10-10

**Authors:** Yaping Ren, Yubin He, Yue Hao, Zhijie Yue, Zixiong Zhu, Xuewen Li

**Affiliations:** Department of Cardiovascular Medicine, Third Hospital of Shanxi Medical University, Shanxi Bethuen Hospital, Shanxi Academy of Medical Sciences, Tongji Shanxi Hospital, No.99 Longcheng street, Xiaodian District, Taiyuan, 030032 Shanxi Province China

**Keywords:** Disability-adjusted life years, Estimated annual percentage change, Global burden of disease, Low environmental temperature, Myocarditis, Social-demographic index, Future forecasting

## Abstract

**Background:**

Myocarditis, a condition associated with an elevated risk of heart failure and sudden cardiac death, has been linked to low-environmental temperature exposure. Despite its significant health implications, there has been very limited research to examine the global burden of myocarditis and its association with cold environmental temperatures. This study aimed to comprehensively analyze the impact of low environmental temperatures on myocarditis-related mortality and disability-adjusted life years (DALYs) over a 30-year period, providing evidence to inform global public health strategies.

**Methods:**

Data from the 2021 Global Burden of Disease (GBD) database were used to evaluate deaths, DALYs, age-standardized mortality rate (ASMR), age-standardized DALYs rate (ASDR), and estimated annual percentage change (EAPC) with 95% uncertainty intervals (95% UI) for low environmental temperature-related myocarditis. Analyses were conducted at global, regional, and national levels from 1990 to 2021 and stratified by sex, age, sociodemographic index (SDI), region, and country. The frontier analyses assessed the impact of epidemiological drivers and SDI on the burden. The nordpred model validated the predictions.

**Results:**

From 1990 to 2021, deaths attributable to low environmental temperature-related myocarditis increased by 32%, rising from 1,405 (95% UI, 1056–1818) in 1990 to 1,855 (95% UI, 1288–2438) in 2021. China reported the highest number of deaths and DALYs, while Romania exhibited the highest ASMR and ASDR. Males were disproportionately affected, and the burden was most pronounced in regions with high-middle and middle SDI. Deaths among individuals over 30 years increased significantly, whereas the ASMR for individuals aged 85 years and above exhibited an inverted V-shaped trend. It is projected that by 2046. Males are expected to experience a greater burden of disability and a higher ASDR compared to females.

**Conclusion:**

Low-environmental temperature exposure contributes significantly to myocarditis-related mortality and DALYs. Targeted interventions to mitigate the effects of cold exposure, particularly in high-burden regions and vulnerable populations, are essential to improve public health outcomes.

**Supplementary Information:**

The online version contains supplementary material available at 10.1186/s12872-025-05209-2.

## Background

Myocarditis, a non-specific inflammatory condition of the myocardium with diverse etiologies, can lead to severe clinical manifestations such as heart failure, chest pain, arrhythmias, and even sudden cardiac death. Globally, approximately 1.8 million individuals are affected by this condition annually [1]. This condition can be triggered by infections, immune responses, pharmaceutical agents, or immunizations [2]. It is predominantly induced by viruses but may also be caused by other infectious agents, including bacteria, protozoa and fungi. Additionally, myocarditis can result from exposure to toxic substances, drugs, and systemic immune-mediated diseases. Many viral infections have a characteristic seasonal distribution. For example, influenza viruses are prevalent during the winter months [3]. The Global Burden of Disease (GBD) 2019 study reported a significant upward trend in myocarditis cases, increasing from 780,410 in 1990 to 1,265,770 in 2019, accompanied by an increase in associated deaths from 19,618 to 32,449 during the same period [4]. This increase has significantly contributed to the global disease burden, particularly in cases of sudden death and dilated cardiomyopathy [5]. Frazi et al. found that 20% of myocarditis cases in individuals under 40 years of age are associated with sudden death, underscoring the critical need for effective management strategies [6]. 

Currently, the treatment for myocarditis lacks specificity, highlighting the importance of comprehensive care and preventive strategies to lower risks and enhance clinical outcomes. The 2021 GBD study identified extreme environmental temperatures as crucial risk factors for myocarditis, offering a foundation for targeted prevention strategies. Epidemiological research has demonstrated a U-shaped or J-shaped relationship between environmental temperature extremes and mortality risk associated with cardiovascular, respiratory, and other diseases [7]. Low environmental temperatures, in particular, have been found to exert a more pronounced impact on health outcomes compared to high environmental temperatures. However, this relationship varies greatly across different geographic regions and populations [8]. 

Recent studies have further explored the impact of environmental temperature extremes on disease burden. Analysis of data from January 1, 1980, to December 31, 2016, found that cold-related disease burden is primarily driven by cardiovascular diseases, chronic respiratory diseases, metabolic diseases, and acute respiratory infections [9]. Additionally, research has shown that from 1990 to 2021, the global disease burden of myocarditis in children aged 0–14 years was influenced by various factors, with low environmental temperature being a key risk factor for myocarditis-related mortality in this age group [10]. Furthermore, the GBD 2021 database analysis indicated that both high and low environmental temperatures increase the risk of myocarditis globally [11]. 

While epidemiological data establish low temperature as a risk factor for myocarditis, the underlying biological mechanisms remain poorly understood. Recent animal studies suggest that cold stress may impair immune defense through glucocorticoid-mediated immunosuppression and oxidative stress. In tropical rodents, cold exposure reduced leukocyte counts and activated stress-responsive chaperones (HSF-1/HSP-70), while melatonin supplementation reversed these effects—highlighting a potential neuroendocrine-immune axis in temperature adaptation. These findings propose testable hypotheses for human myocarditis pathogenesis under cold stress [12]. 

Despite these insights, no systematic, updated, global-level assessment of low-temperature-related myocarditis burden has been conducted using the GBD 2021 dataset. To address this gap, our study quantifies the burden, trends, and regional disparities of myocarditis attributable to low environmental temperatures from 1990 to 2021. Leveraging the most recent and comprehensive GBD data, this analysis aims to inform targeted prevention strategies and refine clinical management in the context of climate change.

## Method

### Data sources and collection

Data on overall Low environmental temperature and its associated myocarditis deaths and DALYs from 1990 to 2021 were obtained from the Global Health Data Exchange query tool (https://ghdx.healthdata.org). This dataset encompasses 204 countries and regions, 359 diseases and injuries, and 84 risk factors. We selected ‘all countries and regions’ or ‘GBD regions’ as locations, ‘all causes’ and ‘myocarditis’ as causes, ‘low environmental temperature’ as the risk factor, ‘DALYs’ and ‘deaths’ as metrics, measured by ‘number’ and ‘rate’. Gender categories included ‘male,’ ‘female’ and ‘both’, with age grouped in 5-y intervals.

The environmental temperature data were obtained from the ERA5 grid produced by the European Centre for Medium-Range Weather Forecasts [9]. While ERA5 provides high-resolution climate data, its gridded format may misclassify localized temperature extremes due to spatial averaging. This could attenuate exposure-response estimates, particularly in topographically diverse regions.

### Assessment of burden

The impact of low environmental temperatures on the burden of myocarditis was evaluated using a comparative risk assessment framework to calculate population attributable fractions (PAFs). Bayesian meta-regression synthesizes heterogeneous data sources via hierarchical priors, while the Gaussian process models spatiotemporal autocorrelation to interpolate missing data. Exposure-response curves were fit using splines to capture non-linear cold effects. These methods provided a robust framework for understanding the contribution of cold exposure to the global burden of myocarditis [10, 11]. 

In GBD 2021, The impact of low environmental temperatures on the burden of myocarditis was derived by a Bayesian meta-regression tool developed by the GBD research team [13, 14]. 

### Statistical analyses

The statistical methodologies used in the 2021 GBD study have been comprehensively detailed in prior research [15]. Age-standardized rates (ASRs) and estimated annual percentage changes (EAPCs) were employed to quantify trends in myocarditis-related DALYs and mortality. ASR measures age-specific rates adjusted according to a standard population distribution and was calculated using the formula:

$$ASR\;=\;\Sigma iAaiwi/\Sigma iAwi\times100,000\;$$ 

where A represents the total number of age groups, a.i. represents the age-specific rate, and wi is the standard population weight for age group i.

EAPC, which reflects the trend of ASR over time, was derived by fitting a regression line to the natural logarithm of ASR (y = ln (ASR)) against calendar year (x), using the equation y = α + βx + e. EAPC was calculated as 100×[exp(β) − 1], with 95% uncertainty intervals (UIs) derived from the linear regression model.

The Sociodemographic Index (SDI), assessed for the period 1950–2019, was used to examine the influence of socio-economic factors on age-standardized rates. The SDI scale ranges from 0 to 1, representing factors such as fertility, education, and income.

The Frontier Study is a method that evaluates how efficiently countries achieve optimal health outcomes. We used frontier analysis to compare each country’s health performance against the global best practices and identify the gap between actual ASRs and theoretical optimal values at different development levels [16]. 

Projections for the disease burden from 2022 to 2044 were made using the Bayesian Age-Period-Cohort (BAPC) model with nested Laplace approximations.

All statistical analyses and graphical visualizations were performed using R version 4.3.2 (R foundation for Statistical Computing, Vienna, Austria).

## Results

### Risk factors for myocarditis

Both low and high environmental temperatures re-identified as primary risk factors for myocarditis, with low environmental temperature being the most significant. GBD data from 1990 to 2021 confirms that low environmental temperature was the leading risk factor for myocarditis throughout this period. In 2021, myocarditis-related deaths and DALYs caused by low-environmental temperature-induced myocarditis accounted for 73.1% and 70.6%, respectively. Conversely, those caused by myocarditis induced by high environmental temperatures accounted for 26.9% and 29.4%, respectively.

The age-standardized death rate and disability rates caused by low- environmental- temperature-induced myocarditis were 73% and 70.1%, respectively. SDI regions were classified into high-SDI, high-middle SDI, middle SDI, low-middle SDI, and low SDI. In 2021, myocarditis-related deaths due to low environmental temperatures accounted for 85.9%, 83.1%, 66.1%, 60%, and 64.5% of total deaths in these regions, respectively. Similarly, the proportion of DALYs attributable to low-environmental-temperature-induced myocarditis was 83.4%, 81.9%, 66.4%, 57.7%, and 65.3% in the respective SDI regions.

**Fig. 1:**
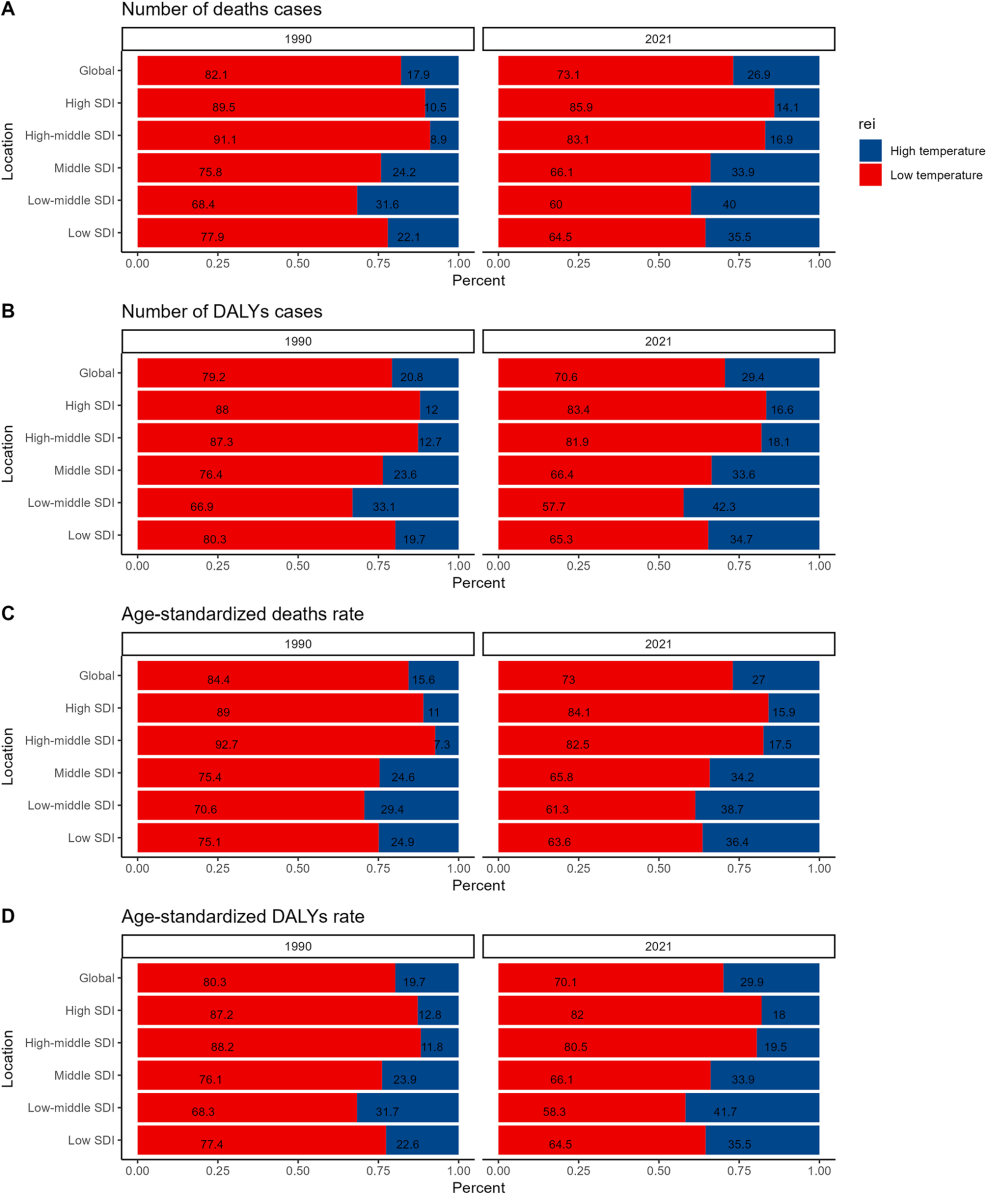
Percentage contributions of major risk factors to number of deaths, DALYs, age-standardized death and DALYs rate of myocarditis, 1990–2021.DALYs: disability-adjusted life years

Additionally, the age-standardized death rates associated with low-environmental-temperature-induced myocarditis in these regions were 84.1%, 82.5%, 65.8%, 61.3%, and 63.6%, while the age-standardized DALY rates were 82%, 80.5%, 66.1%, 58.3%, and 64.5%. The findings indicate that high-SDI regions experienced the greatest burden of low- environmental-temperature-related myocarditis, with deaths and DALYs exceeding 80% (Fig. 1).

### Global estimated data for hypothermia-related myocarditis in 1990 and 2021

In 1990, hypothermia-induced myocarditis caused 1,405 deaths globally (95% UI, 1056 — 1818). By 2021, this number had increased to 1,855 deaths (95% UI, 1288 — 2438). The global age-standardized mortality rate (ASMR) for hypothermia-related myocarditis decreased from 0.04 per 100,000 (95% UI, 0.03 — 0.05) in 1990 to 0.02 per 100,000 (95% UI, 0.02 — 0.03) in 2021 (Table 1), indicating an overall decline over the past three decades.

**Table 1:** Deaths cases and age-standardized rate of Low-temperature-related myocarditis and their EAPCs from 1990 to 2021 at the sex, age, SDI region

	No.1990(95UI)	ASMR.1990(95%UI)	No.2021(95%UI)	ASMR.2021(95%UI)	EAPC(%)(95%UI)
Global	1405 (1056–1818)	0.04 (0.03–0.05)	1855 (1288–2438)	0.02 (0.02–0.03)	−1.98 (−2.44–1.51)
Gender					
Female	704 (498–938)	0.03 (0.02–0.05)	877 (589–1152)	0.02 (0.01–0.03)	−2.26 (−2.79–1.73)
Male	701 (532–959)	0.04 (0.03–0.05)	977 (680–1321)	0.03 (0.02–0.04)	−1.66 (−2.05–1.27)
Age					
< 5 years	326 (205–496)	0.05 (0.03–0.08)	89 (55–137)	0.01 (0.01–0.02)	−4.25 (−4.44–4.06)
5–9 years	35 (26–50)	0.01 (0-0.01)	17 (12–25)	0 (0–0)	−3.15 (−3.43–2.87)
10–14 years	24 (17–32)	0 (0-0.01)	16 (11–21)	0 (0–0)	−2.29 (−2.64–1.95)
15–19 years	36 (26–49)	0.01 (0-0.01)	22 (15–31)	0 (0-0.01)	−2.29 (−2.7–1.89)
20–24 years	38 (28–54)	0.01 (0.01–0.01)	29 (19–40)	0 (0-0.01)	−1.47 (−1.78–1.16)
25–29 years	35 (26–49)	0.01 (0.01–0.01)	31 (22–44)	0.01 (0-0.01)	−1.31 (−1.6–1.03)
30–34 years	35 (25–50)	0.01 (0.01–0.01)	41 (29–57)	0.01 (0-0.01)	−1.16 (−1.48–0.85)
35–39 years	43 (31–60)	0.01 (0.01–0.02)	51 (35–69)	0.01 (0.01–0.01)	−1.38 (−1.71–1.06)
40–44 years	43 (31–60)	0.02 (0.01–0.02)	58 (41–79)	0.01 (0.01–0.02)	−1.22 (−1.53–0.91)
45–49 years	42 (29–60)	0.02 (0.01–0.03)	68 (50–92)	0.01 (0.01–0.02)	−0.95 (−1.24–0.66)
50–54 years	45 (31–63)	0.02 (0.01–0.03)	81 (58–108)	0.02 (0.01–0.02)	−0.97 (−1.28–0.65)
55–59 years	51 (36–70)	0.03 (0.02–0.04)	96 (63–127)	0.02 (0.02–0.03)	−0.93 (−1.2–0.65)
60–64 years	57 (40–77)	0.04 (0.03–0.05)	90 (65–118)	0.03 (0.02–0.04)	−0.95 (−1.22–0.68)
65–69 years	65 (47–90)	0.05 (0.04–0.07)	114 (78–152)	0.04 (0.03–0.06)	−1.13 (−1.44–0.82)
70–74 years	65 (47–90)	0.08 (0.06–0.11)	131 (88–173)	0.06 (0.04–0.08)	−1.09 (−1.55–0.63)
75–79 years	89 (67–115)	0.14 (0.11–0.19)	147 (92–197)	0.11 (0.07–0.15)	−1.16 (−1.61–0.7)
80–84 years	125 (92–157)	0.35 (0.26–0.45)	217 (143–290)	0.25 (0.16–0.33)	−1.62 (−2.12–1.12)
85–89 years	135 (96–166)	0.89 (0.64–1.1)	255 (163–338)	0.56 (0.36–0.74)	−2.31 (−2.9–1.72)
90–94 years	86 (59–107)	2.01 (1.39–2.49)	204 (127–270)	1.14 (0.71–1.51)	−2.63 (−3.51–1.73)
95 + years	30 (20–39)	2.99 (1.96–3.82)	96 (58–126)	1.76 (1.06–2.31)	−2.34 (−3.29–1.38)
SDI region					
High-middle SDI	552 (405–662)	0.08 (0.06–0.1)	700 (478–866)	0.04 (0.03–0.05)	−3.17 (−3.92–2.42)
High SDI	177 (136–206)	0.02 (0.01–0.02)	239 (189–273)	0.01 (0.01–0.02)	−1.36 (−1.91–0.8)
Low-middle SDI	132 (45–258)	0.02 (0.01–0.03)	194 (73–363)	0.01 (0.01–0.03)	−0.58 (−0.83–0.33)
Low SDI	47 (18–92)	0.01 (0-0.03)	63 (24–131)	0.01 (0-0.02)	−0.85 (−1.2–0.5)
Middle SDI	496 (362–676)	0.04 (0.03–0.06)	655 (372–956)	0.03 (0.02–0.04)	−1.17 (−1.52–0.83)

*No* number, *ASMR* age-standardized deaths rate, *EAPC* Estimated annual percent change

* 95%UI:95%uncertainty interval*

*SDI Socio-Demographic Index. GBD: Global Burden of Disease*

In 1990, the estimated DALYs for hypothermia-related myocarditis were 62,552 (95% UI 46193–85513) with an age-standardized DALYs rate (ASDR) of 1.22 per 100,000 (95% UI, 0.91–1.63). By 2021, these values had decreased to 51,286 DALYs (95% UI, 36675–68735), and an ASDR of 0.65 per 100,000 (95% UI, 0.47–0.88) (Table 2).

**Table 2:** DALYs cases and age-standardized rate of Low-temperature-related myocarditis and their EAPCs from 1990 to 2021 at the sex, age, SDI region

	No.1990(95%UI)	ASDR.1990(95%UI)	No.2021(95%UI)	ASDR.2021(95%UI)	EAPC(%)(95%UI)
Global	62,552 (46193–85513)	1.22 (0.91–1.63)	51,286 (36675–68735)	0.65 (0.47–0.88)	−2.31 (−2.56–2.06)
Gender					
Female	28,183 (19160–40567)	1.09 (0.75–1.54)	21,175 (14242–28699)	0.52 (0.35–0.72)	−2.53 (−2.83–2.24)
Male	34,369 (24725–49238)	1.35 (1-1.9)	30,111 (21318–40743)	0.79 (0.56–1.07)	−2.11 (−2.33–1.89)
Age					
< 5 years	28,995 (18208–44087)	4.68 (2.94–7.11)	7952 (4889–12248)	1.21 (0.74–1.86)	−4.25 (−4.43–4.06)
5–9 years	2937 (2169–4136)	0.5 (0.37–0.71)	1423 (955–2034)	0.21 (0.14–0.3)	−3.15 (−3.43–2.87)
10–14 years	1828 (1337–2507)	0.34 (0.25–0.47)	1207 (817–1663)	0.18 (0.12–0.25)	−2.3 (−2.64–1.96)
15–19 years	2594 (1874–3586)	0.5 (0.36–0.69)	1607 (1111–2264)	0.26 (0.18–0.36)	−2.3 (−2.7–1.89)
20–24 years	2601 (1903–3626)	0.53 (0.39–0.74)	1945 (1293–2708)	0.33 (0.22–0.45)	−1.48 (−1.79–1.17)
25–29 years	2215 (1601–3072)	0.5 (0.36–0.69)	1956 (1354–2760)	0.33 (0.23–0.47)	−1.32 (−1.6–1.03)
30–34 years	2028 (1454–2893)	0.53 (0.38–0.75)	2390 (1682–3280)	0.4 (0.28–0.54)	−1.16 (−1.47–0.85)
35–39 years	2265 (1614–3145)	0.64 (0.46–0.89)	2687 (1844–3645)	0.48 (0.33–0.65)	−1.38 (−1.71–1.06)
40–44 years	2068 (1486–2885)	0.72 (0.52–1.01)	2780 (1950–3786)	0.56 (0.39–0.76)	−1.23 (−1.54–0.92)
45–49 years	1801 (1253–2573)	0.78 (0.54–1.11)	2939 (2128–3973)	0.62 (0.45–0.84)	−0.95 (−1.24–0.66)
50–54 years	1702 (1184–2411)	0.8 (0.56–1.13)	3083 (2198–4106)	0.69 (0.49–0.92)	−0.97 (−1.28–0.65)
55–59 years	1693 (1212–2350)	0.91 (0.65–1.27)	3206 (2101–4249)	0.81 (0.53–1.07)	−0.93 (−1.2–0.65)
60–64 years	1633 (1165–2224)	1.02 (0.73–1.38)	2606 (1873–3413)	0.81 (0.59–1.07)	−0.95 (−1.22–0.68)
65–69 years	1585 (1145–2191)	1.28 (0.93–1.77)	2781 (1906–3694)	1.01 (0.69–1.34)	−1.13 (−1.43–0.82)
70–74 years	1294 (938–1798)	1.53 (1.11–2.12)	2611 (1767–3450)	1.27 (0.86–1.68)	−1.1 (−1.55–0.64)
75–79 years	1414 (1063–1835)	2.3 (1.73–2.98)	2341 (1471–3140)	1.77 (1.12–2.38)	−1.17 (−1.63–0.71)
80–84 years	1567 (1148–1971)	4.43 (3.25–5.57)	2704 (1786–3610)	3.09 (2.04–4.12)	−1.63 (−2.13–1.12)
85–89 years	1339 (954–1653)	8.86 (6.31–10.94)	2529 (1614–3347)	5.53 (3.53–7.32)	−2.32 (−2.9–1.73)
90–94 years	744 (513–921)	17.37 (11.97–21.5)	1759 (1098–2332)	9.83 (6.14–13.03)	−2.63 (−3.51–1.73)
95 + years	249 (164–318)	24.49 (16.06–31.26)	780 (470–1020)	14.31 (8.63–18.72)	−2.37 (−3.32–1.42)
SDI region					
High-middle SDI	17,129 (12625–21579)	1.96 (1.41–2.44)	15,614 (10749–19755)	1.02 (0.7–1.29)	−2.84 (−3.25–2.43)
High SDI	7469 (5761–8757)	0.91 (0.69–1.07)	6691 (5324–7656)	0.58 (0.47–0.68)	−1.74 (−2.26–1.22)
Low-middle SDI	7332 (2399–15153)	0.63 (0.21–1.24)	8426 (2992–15750)	0.48 (0.17–0.89)	−0.66 (−0.92–0.4)
Low SDI	2826 (952–5651)	0.52 (0.19–1.01)	3127 (1328–5934)	0.33 (0.12–0.67)	−1.53 (−1.83–1.23)
Middle SDI	27,735 (19218–39065)	1.67 (1.19–2.31)	17,339 (10725–24595)	0.76 (0.48–1.08)	−2.71 (−2.94–2.47)

*No *number *ASDR* age-standardized DALYs rate,* EAPC* Estimated annual percent change

* 95%UI:95%uncertainty interval. *

*SDI Socio-Demographic Index, GBD Global Burden of Disease*

**Fig. 2:**
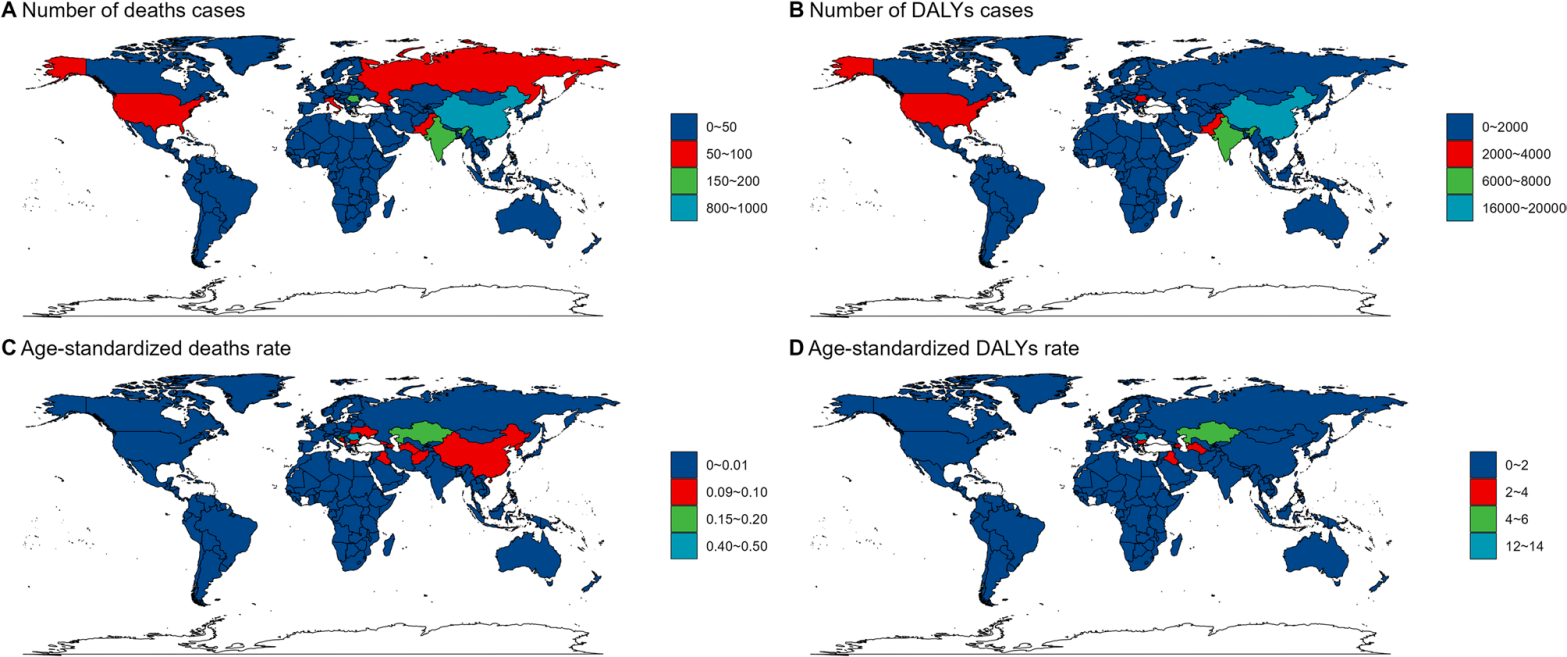
Low-temperature-related myocarditis in 204 countries and territories worldwide.** A** number of deaths numbers in 2021; **B** number of DALYs cases in 2021; **C** age-standardized deaths rate in 2021; **D** age-standardized DALYs rate in 2021 DALYs: disability-adjusted life years

Among 204 countries, China recorded the highest number of deaths (818; 95% UI, 409–1186) and DALYs (19,460; 95% UI, 10006–27376) from low-environmental- temperature-related myocarditis in 2021. However, Romania had the highest ASMR (0.3 per 100,000; 95% UI: 0.22–0.42) and ASDR (0.48 per 100,000; 95% UI: 0.34–0.63) globally in the same year (Fig. 2, Supplementary Tables 1 and 2 online). Kazakhstan exhibited the largest increase in EAPC, at 13.61% (11.45–15.81) for ASMR and 12.55% (10.43–14.71) for ASDR, while Italy showed the most significant declines, with EAPC values of −8.77% (−10.42–7.09) for ASMR and ASDR. Regionally, Asia recorded the highest number of deaths and disabilities in 1990, with 850 deaths (95% UI, 585–1229) and 46,713 DALYs (95% UI, 32213–67336) (Supplementary Table 1, Supplementary Table 2 online). However, by 2021, these figures had risen to 1,225 deaths (95% UI: 782–1722) and 35,609 disabilities (95% UI: 24069–50465). Central Europe led in ASMR (2.59 per 100,000; 95% UI: 2.06–3.09) and ASDR (3.14 per 100,000; 95% UI: 2.09–4.15) in 2021.

Central Asia experienced the largest increase in EAPC for ASMR (2.1%; 95% UI: 1.43–2.76) and ASDR (1.91%; 95% UI: 1.18–2.65), while Western Africa showed the most pronounced declines, with EAPC values of −5.43% (95% UI: −6.07 – −4.78) (Supplementary Tables 1 and 2, Supplementary Fig. 1 online).

In terms of age distribution, deaths were primarily concentrated in the 85–90 age group, where DALYs were concentrated in the 55–59 age group. Changes in age-specific rates revealed an upward trend, with the highest values recorded in individuals aged 95 years and older (Supplementary Fig. 2 online).

According to SDI classification, ASMR and ASDR peaked in the high-middle SDI category in 2021, showing the largest increases over the 30-year period. The lowest ASMR and ASDR were in the low SDI category, showing a marked decline. The number of deaths was most prevalent in the high-middle SDI category, whereas DALYs were most frequently observed in the middle-SDI category (Supplementary Fig. 3 online).

In 2021, males exhibited the highest rates of deaths, disabilities, ASMR, and ASDR due to low-environmental-temperature-induced myocarditis, highlighting a notable gender disparity in disease burden (Supplementary Fig. 4 online).

### Global trends in hypothermia-related myocarditis from 1990 to 2021

In 2021, an estimated 1,855 deaths (95% UI, 1288–2438) were attributed to hypothermia-related myocarditis globally, representing a 32% increase since 1990. The EAPC for the ASMR was − 1.98 (95% UI, −2.44–1.51) (Table 1). According to the diagram, the ASMR declined from 0.04 to 0.02 per 100,000 during the period from 2003 to 2005. Despite this decline, the ASMR in this period remained higher compared to that in 1990. Subsequently, the ASMR exhibited a significant decrease, deviating from the overall trend (Supplementary Fig. 5 online). Age and sex were key factors influencing the ASMR.

DALYs associated with hypothermia-related myocarditis totaled 51,286 (95% UI: 36675–68735) in 2021, representing a 17.2% reduction compared to 1990. Similarly, the age-standardized DALY rate (ASDR) decreased from 1.22 to 0.65 per 100,000 during the same period (Table 2). The DALYs decreased by 0.18% from 62,552 (95% UI, 46193–85513) in 1990 to 51,286 (95% UI, 36675–68735) in 2021. The ASDR dropped from 1.22 per 100,000 (95% UI, 0.911.63) in 1990 to 0.65 per 100,000 (95% UI, 0.47–0.88) in 2021 (Table 2). Additionally, the ASDR exhibited a notable decline from 2008 to 2021, diverging from the general trend (Supplementary Fig. 5 online).

Among the 21 regions analyzed in the GBD study, Central Asia exhibited the most significant increases in ASMR and ASDR for hypothermia-related myocarditis between 1990 and 2021, with an ASMR increase of 2.1% (95% UI 1.43–2.76) and an ASDR increase of 1.91% (95% UI 1.18–2.65). In contrast, Central Europe recorded the lowest ASMR change (−5.43%, 95% UI, −6.07–4.78), while Andean Latin America had the lowest ASDR (−5.05%, 95% UI −5.35–4.74) (Tables 1 and 2).

**Fig. 3:**
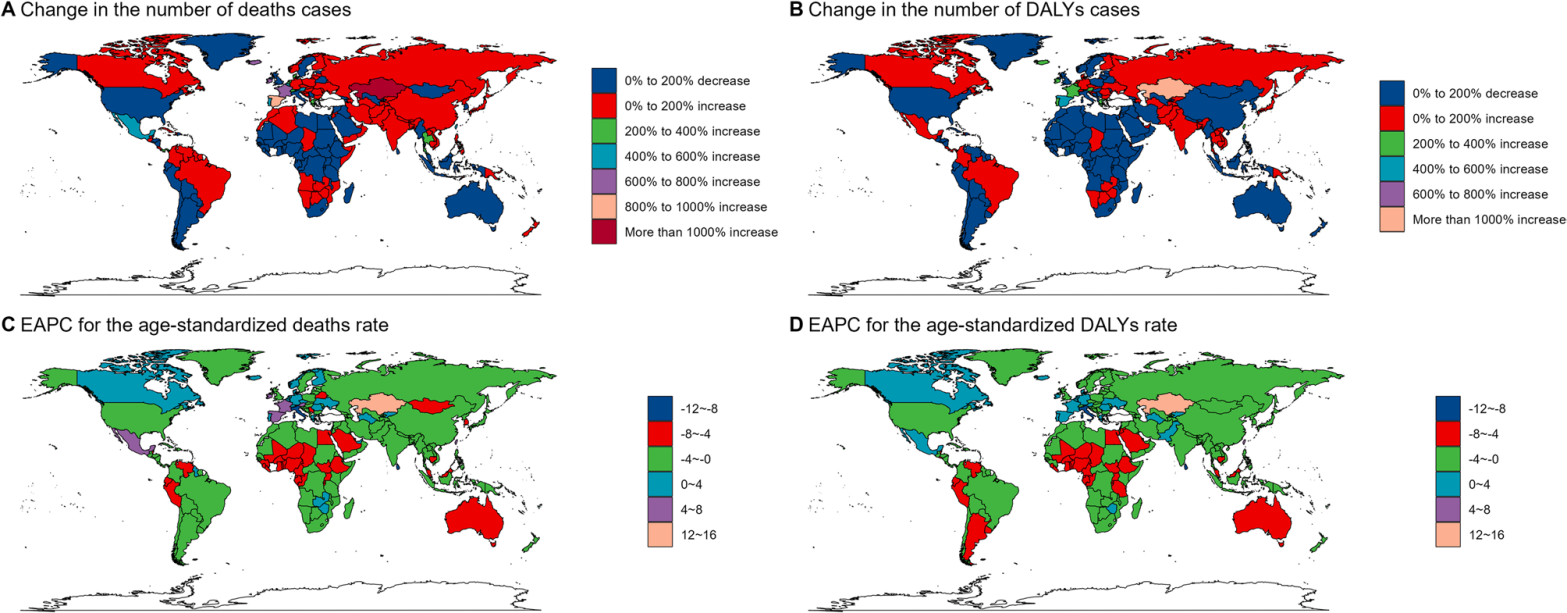
Global trends in hypothermia-related myocarditis from 1990 to 2021. **A** Changes in the number of deaths cases from 1990–2021; **B** Changes in the number of DALYs cases from 1990–2021;** C** EAPC analysis of age-standardized deaths rate from 1990–2021; **D** EAPC analysis of age-standardized DALYs rate from 1990–2021 DALYs: disability-adjusted life years; EAPC: Estimated annual percent change

At the country level, Kazakhstan experienced the most substantial increases in mortality, DALYs, ASMR (13.61%, 95% UI: 11.45–15.81), and ASDR (12.55%, 95% UI: 10.43–14.71) associated with hypothermia-related myocarditis from 1990 to 2021. Conversely, Italy had the lowest ASMR (−8.77%, 95% UI, −10.42–7.09) and ASDR (−9.04%, 95% UI −10.62–7.44). Over the past three decades, Kazakhstan, Spain, France, and Portugal showed the largest increases in the burden of myocarditis, exceeding 200% and, in some cases, surpassing 400%. By comparison, the smallest increases in disease burden were predominantly observed in Africa (Fig. 3; Supplementary Tables 1, 2 online).

A gender-based analysis indicated that males exhibited significantly higher DALYs, ASMR, and ASDR associated with hypothermia-related myocarditis compared to females. Male deaths increased nearly 39.4%, 1.60 times the rise observed in females. For DALYs, male cases declined by 12.4%, representing half the increase noted in females (Supplementary Fig. 6 online).

According to classifications based on the SDI, the burden of hypothermia-related myocarditis varied across regions. High-middle and middle SDI regions experienced the greatest impact. In the high-middle SDI region, the ASMR dropped from 0.08 per 100,000 (95% UI, 0.06–0.1) in 1990 to 0.04 per 100,000 (95% UI, 0.03–0.05) in 2021. In the middle SDI region, the ASMR declined from 0.04 per 100,000 to 0.03 per 100,000 over the same period. The middle-SDI region had the highest DALYs, while the high-middle SDI group exhibited the highest ASDR (−2.84%, 95% UI, −3.25–2.43) (Supplementary Fig. 7 online).

Age-stratified data revealed a significant rise in deaths among individuals older than 30 years, while those under 5 years of age experienced a marked decrease in both deaths and DALYs. Among individuals aged 5 to 30 years, mortality rates remained relatively stable with minor fluctuations, and changes in DALYs were minimal. ASMR and ASDR showed an inverted V-shape trend for individuals aged 85 years and above, initially rising before declining, while remaining stable for those under 85 years (Supplementary Fig. 8 online).

From 1990 to 2021, the 55–59 age group exhibited the highest EAPC in ASRs at −0.93% (95% UI, −1.2–0.65). The most substantial increase in deaths occurred in the 85–89 age group, with a rise of approximately 88.8%. The largest increase in DALYs was observed in 55–59 age group, with an approximate rise of 88.2%. T Individuals aged 95 years and older demonstrated the highest reductions in ASMR (−2.34%, 95% UI, −2.39–2.18) and ASDR (−2.37%, 95% UI, −3.32–1.42) (Supplementary Fig. 8; Tables 1 and 2 online).

### Frontier analysis of the association between deaths and DALYs due to hypothermia-related myocarditis and the level of national development

**Fig. 4:**
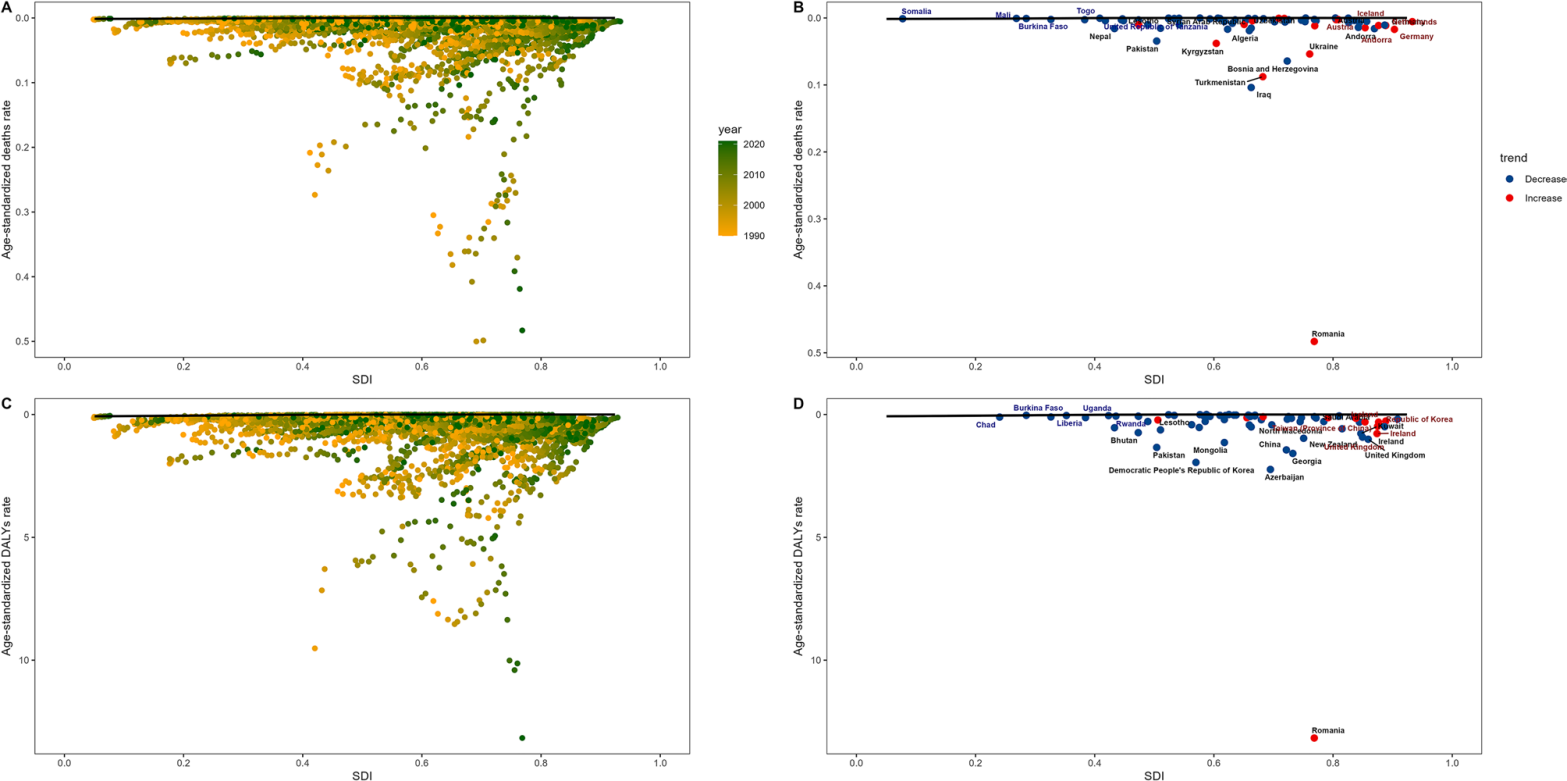
Frontier analysis of the association between deaths and DALYs from hypothermia-related myocarditis and the level of national development DALYs: disability-adjusted life years

The analysis revealed that between 1990 and 2021, the disparities between the actual values of ASMR and ASDR and the theoretical optimal performance widened with the increase in SDI (Figs. 4A, B). In the ASMR analysis, countries with an SDI range of 0.6 to 0.9, such as Romania, Andorra, Kyrgyzstan, and Turkmenistan showed an increasing gap from the theoretical optimal performance, and this gap was at a higher level. Conversely, a limited number of countries, such as Iraq, Bosnia and Herzegovina, and Pakistan, exhibited a reduction in the gap.

In the ASDR analysis, countries such as Romania experienced a significant widening of the gap and ranked among the highest in terms of disparity. However, in most countries, including Georgia, New Zealand, Pakistan, and Azerbaijan, a reduction in the gap was observed. (Figs. 4C, D)

### Forecast of the overall burden of low temperature infectious

**Fig. 5:**
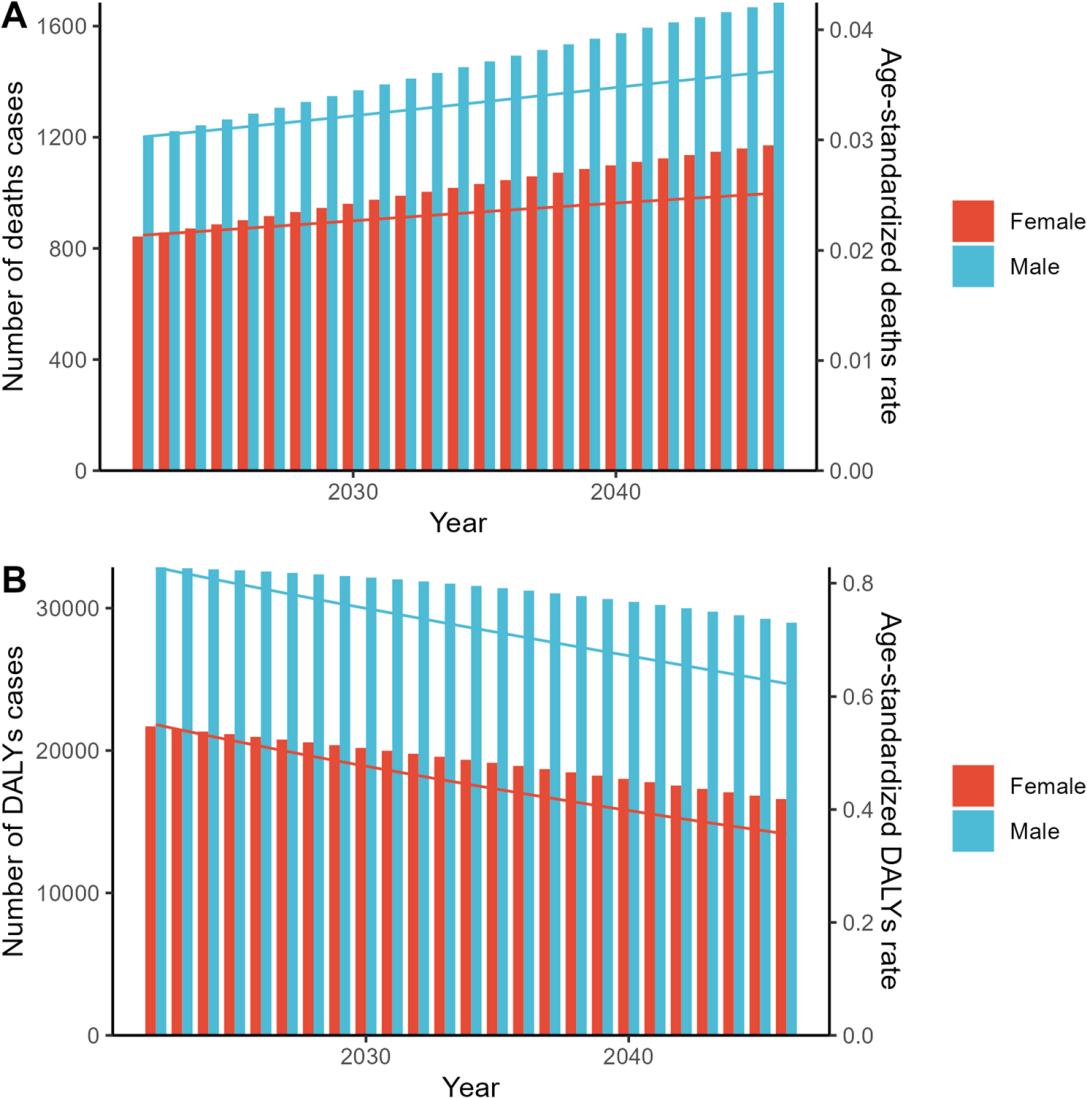
Utilize the BAPC model to predict the number of deaths and DALYs, age-standardized rates (per 100 000 persons) of deaths and DALYs associated with low temperature related to myocarditis for different genders. Abbreviations: BAPC, Bayesian age-period-cohort analysis (BAPC). DALYs: disability-adjusted life years

According to the Nordpred model, it is projected that by 2046, low environmental temperature-related myocarditis will result in 1,171 deaths and 16,594 DALYs globally. The number of deaths is expected to gradually increase from 1990 to 2046, with males consistently exhibiting a higher age-standardized mortality rate (ASMR). However, DALYs are projected to decline, with an age-standardized disability rate (ASDR) of 0.36 per 100,000. Males are expected to experience a greater burden of disability and a higher ASDR compared to females (Fig. 5, Supplementary Table 3).

### Correlation analysis

**Fig. 6:**
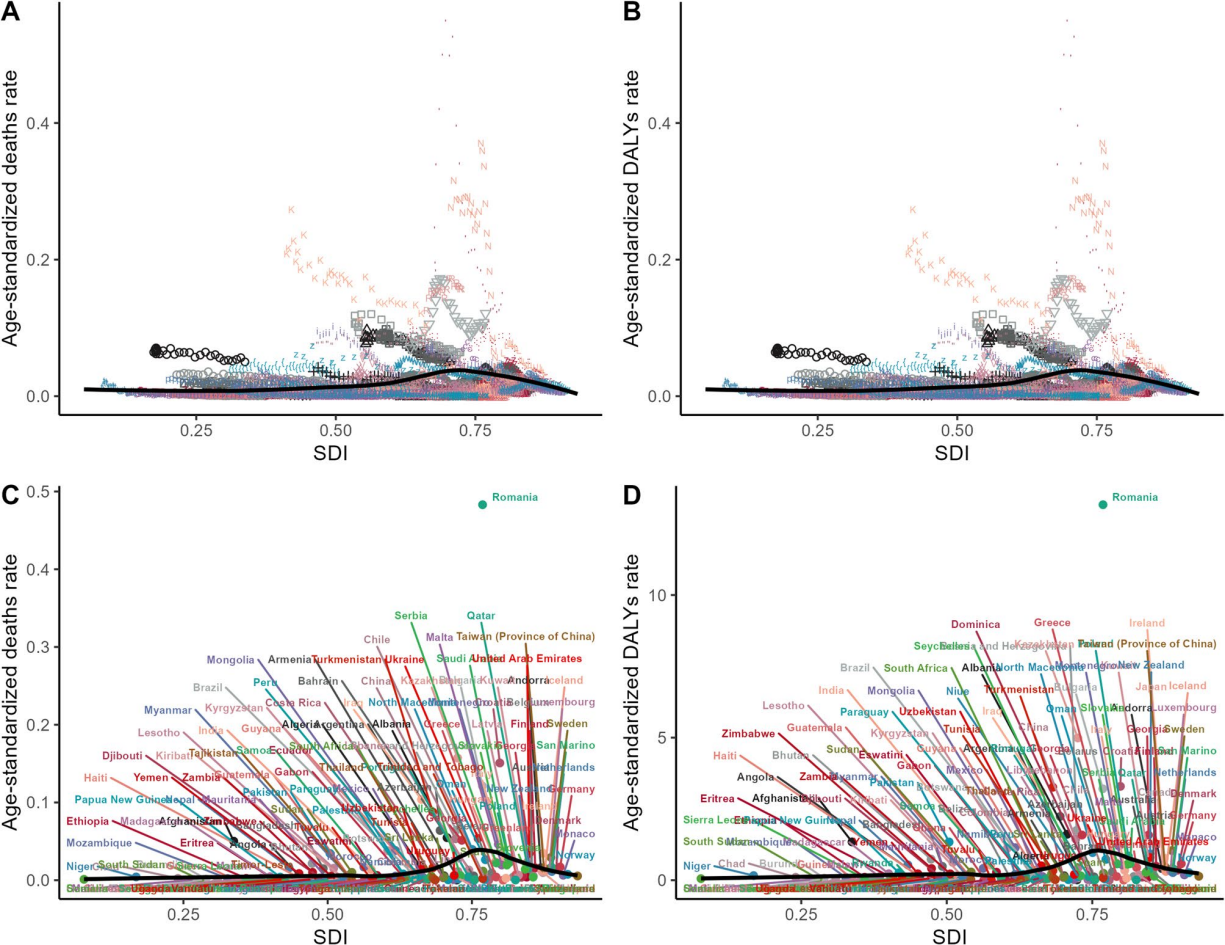
The correlation between SDI and ASR of low temperature related to myocarditis 2021. age-standardized death rate **A**, **C** and age-standardized DALYs rate **B**, **D**. DALYs: disability-adjusted life years; SDI: sociodemographic index

As the SDI increased, the ASMR and ASDR of low-temperature-related myocarditis generally showed a trend of slow rise followed by a rapid decline (Fig. 6). This increasing trend was pronounced when the SDI value was within the range of 0.5–0.75; however, once the SDI exceeded 0.75, the corresponding indicators decreased significantly. This suggests that countries with higher SDI tend to report a lower burden of low-temperature-related myocarditis. Moreover, Romania clearly shows significantly higher ASMR and ASDR.

## Discussion

This study highlights the significant global burden of myocarditis associated with low environmental temperatures, emphasizing its prevalence as a primary risk factor. Our findings confirm that low-environmental-temperature-induced myocarditis was responsible for 73.1% of myocarditis-related deaths and 70.6% of DALYs in 2021, with a notable prevalence in high-SDI regions. Over the past three decades, the global age-standardized mortality rate (ASMR) and disability-adjusted life years (DALYs) associated with hypothermia-related myocarditis have decreased, but the burden remains substantial, especially in certain regions like Central Asia and Eastern Europe. The study also identifies a gender disparity, with males exhibiting a higher burden of disease. This finding aligns with established patterns of viral infections, where men demonstrate both greater susceptibility to pathogens like COVID-19 and worse clinical outcomes compared to women - a phenomenon likely mediated by fundamental sex-based differences in immune responses that may similarly influence the severity of virus-associated heart disease [3]. Additionally, while there have been regional improvements, such as in Italy, countries like Kazakhstan have seen significant increases in mortality and DALYs, indicating a rising challenge in some regions.

This study represents the first comprehensive assessment of myocarditis risk factors from 1990 to 2021, with a particular focus on the influence of ambient temperature. The findings indicate that low environmental temperatures significantly impact the global burden of myocarditis. A report published by The Lancet highlights climate change as a major health threat, attributing this to the increased frequency of extreme heatwaves and cold snaps, which directly affect human health and heighten the risk of exposure. According to the GBD 2019 report, 1.69 million deaths worldwide were associated with suboptimal temperatures, with low environmental temperatures contributing more substantially to mortality than high environmental temperatures [17]. A multi-country study reported that the mortality rate attributable to low environmental temperatures (7.29%) far exceeded that from high environmental temperatures (0.42%) [18]. These findings align with previous research, emphasizing the significant health implications of climate change. Although high environmental temperatures often garner attention, this study, alongside others, underscores the severe health risks posed by low environmental temperatures.

Hypothermia, characterized by abnormally low body temperature, presents a stark contrast to hyperthermia, in which metabolism and body functions become dysregulated. Severe hypothermia is a life-threatening condition associated with atrial and ventricular dysrhythmias, coagulopathy, cardiac, and central nervous system depression [19]. During hypothermia, spontaneous circulation is significantly reduced, and with prolonged or deep cooling, circulatory failure may develop, which limits the potential for safe recovery [20]. 

Experimental studies utilizing invasive hemodynamic monitoring techniques in intact animal models, including dogs, rats, mice, and pigs, have consistently demonstrated that hypothermia directly affects myocardial excitation-contraction coupling and actin-myosin interactions, thereby disrupting normal cardiac function [21]. 

The study identified China as having the highest number of deaths and DALYs attributable to low-environmental-temperature-related myocarditis, consistent with our prior research. A study analyzing data from 1990 to 2019 indicated that while the overall mortality rate from cardiovascular diseases (CVD) associated with non-optimal temperatures in China has declined, the burden caused specifically by low temperatures is particularly severe [22]. 

Over the past two decades, the People’s Republic of China has experienced two large-scale severe acute respiratory syndrome (SARS) epidemics, both occurring during winter. This season, characterized by cold and dry conditions, provides an environment more favorable for viral survival and transmission compared to cold temperatures alone [23, 24]. During severe winters, dried viral particles in the air form a dangerous viral form that can survive in air currents for extended periods [25]. Cold winter conditions not only enhance viral survival and spread but also weaken human innate immunity. Cold temperatures lead to a reduction in blood supply and thus a decrease in the supply of immune cells to the nasal mucosa. Additionally, low humidity can reduce the ability of the ciliated cells in the airways to remove viral particles, secrete mucus, and repair the cells of the airways.

In such conditions, signaling proteins released by human cells to alert neighboring cells of viral infections are also less effective, further compromising the innate immune defense system [26]. Studies from India and Spain similarly indicate that low environmental temperatures present a higher attributable risk for mortality compared to high environmental temperatures [27]. These findings are crucial for countries to recognize the health risks associated with ambient environmental temperatures. Targeted interventions should be developed and incorporated into national and regional climate adaptation strategies to mitigate the effects of cold weather, such as year-round planning, winter preparedness programs, cold spell warnings, and emergency response protocols. Conducting vulnerability assessments at community and individual levels can help identify at-risk populations and guide efforts to mitigate the adverse health impacts of cold weather.

Research has demonstrated that low environmental temperatures significantly impact the health of the Romanian population. Romania’s ASMR and ASDR for myocarditis rank among the highest globally, a situation further aggravated by the country’s harsh winter climate. An in-depth study reveals that 13.9% of the Romanian population suffers from moderate or severe food insecurity, with malnutrition affecting 6.6% of children under five years of age [28]. This prevalence of malnutrition is particularly concerning due to its association with an elevated risk of viral myocarditis. Malnutrition leads to the deterioration of cardiac function, impairs immune responses, and increases susceptibility to viral infections, all of which are recognized as contributing factors in the development of viral myocarditis.

Consequently, in order to effectively prevent heart diseases such as viral myocarditis, it is essential to maintain a balanced diet and adequate nutritional intake to maintain an optimal state of health. Efforts to improve the nutritional status and overall lifestyle of the Romanian population should prioritize enhancing the nutritional quality of consumed foods [29]. 

However, while Romania exhibited the highest ASMR and ASDR for low-temperature-related myocarditis and the population’s dietary inadequacies are concerning, the current evidence does not clearly establish a direct causal link between poor nutrition and cold-induced myocarditis specifically. The observed correlation may reflect a broader vulnerability to infectious diseases, including viral myocarditis, rather than a temperature-specific effect. Therefore, while dietary improvements remain a vital public health strategy, more targeted research is needed to elucidate the specific mechanisms by which nutritional status interacts with cold exposure in contributing to myocarditis risk.

Current research indicates that the daily consumption of vegetables and fruits by the Romanian population is significantly below recommended levels. Nutrition guidelines suggest three servings of vegetables and two servings of fruits per day; however, most individuals report consuming only one serving of these food groups daily. Additionally, the consumption of fish and seafood is notably low, with the majority of respondents stating that these foods are rarely or never included in their diet. Nutritionists recommend consuming two to three servings of fish or seafood per week to meet essential nutrient and mineral requirements [30]. Malnutrition in Romania likely elevates general susceptibility to infections, including those triggering myocarditis, rather than directly mediating cold-specific pathways. Socioeconomic interventions targeting nutritional deficits could thus broadly reduce cardiovascular morbidity. The observed challenges may be attributed to insufficient medical resources and the lack of effective preventive measures. To address this urgent problem, the Romanian government must implement protection strategies.

Between 1990 and 2021, Kazakhstan showed significant increases in deaths, DALYs, ASMR, and ASDR related to hypothermia-induced myocarditis compared to other countries. Kazakhstan, a large Central Asian nation, encompasses diverse climates and vegetation zones that create favorable environments for various pathogens. Additionally, with its rich variety of animal species, Kazakhstan is a region with a high incidence of infectious diseases, such as rabies, brucellosis, tick-borne encephalitis virus (TBEV) of Kazakhstan [31], and foot-and-mouth diseases [32]. Viral or bacterial infections are recognized as primary causes of myocarditis in this area. Timely development of intervention strategies and vaccine formulations tailored to the genetic diversity of the local pathogen strains is essential.

Globally, the burden of low-environmental-temperature-related myocarditis reached a turning point in 2005, with increasing deaths, ASMR, and ASDR observed until that year, followed by a decline. This trend may be attributed to improvements in the diagnosis and treatment of myocarditis [33]. Regions with high to middle SDI typically show higher ASMR and ASDR for myocarditis. These findings provide valuable evidence for improving the prognosis and treatment of low-environmental- temperature-related myocarditis and support efforts to mitigate its global burden.

Significant gender disparities are evident in mortality related to cold-related myocarditis, with males experiencing a disproportionately higher burden. The reasons for these differences remain unclear. Evidence suggests that females generally exhibit stronger immune responses to both self-antigens and foreign antigens compared to males, contributing to the unequal distribution of autoimmune and infectious diseases between sexes. Studies in animal models and humans indicate that male individuals are more susceptible to bacterial infections than their female counterparts [34]. 

Additionally, some researchers suggest these differences may arise from factors such as physiological differences, thermoregulation, socioeconomic influences, and treatment outcomes [35, 36]. More prospective studies and comprehensive analyses are required to better understand these differences. These findings are consistent with previous research indicating a prevalence ratio of 1:1.5 to 1:1.7 for myocarditis between women and men [37]. 

A notable decline in deaths and DALYs among children under five years of age has been observed, attributable to targeted investments in infant and child health. These efforts have effectively reduced low-environmental-temperature-related myocarditis, which is linked to 16% to 20% of sudden infant death syndrome cases [38]. 

The elderly population demonstrates greater vulnerability to the effects of low environmental temperatures compared to younger individuals, consistent with prior studies [39–41]. 

This vulnerability may be attributed to aging, as older individuals lose their ability to regulate body temperature, experience physiological decline, and become more susceptible to chronic diseases [42]. 

To mitigate this burden. enhancing prevention and treatment strategies in cold environments is essential. This includes improving medical care. raising public awareness about symptoms. risk factors. and preventive measures. and ensuring timely access to healthcare. Preventive actions should prioritize providing adequate clothing. shelter. and access to medical facilities during cold periods. Additionally. strengthening climate monitoring and early warning systems can help public health authorities take proactive measures to reduce the impact of low environmental temperatures on myocarditis. Given melatonin’s potential role in alleviating cold stress-induced immunosuppression (as observed in animal models), its exploration as an adjuvant therapy for seasonal myocarditis warrants further investigation [12]. 

Although cold exposure remains a primary risk, climate change may alter myocarditis patterns by increasing heatwave frequency (potentially exacerbating cardiac stress) while reducing winter severity in some regions. These dual dynamics warrant longitudinal monitoring. With global warming, the epidemiological patterns of temperature-related myocarditis may shift significantly. While this study focuses on cold exposure, climate change is projected to increase both heatwaves and cold spells, rather than eliminate cold risk. Vulnerable populations may remain disproportionately affected by cold due to socio-economic and infrastructural barriers. Meanwhile, high temperatures have also been linked to cardiovascular stress and inflammation, potentially increasing myocarditis risk. Burkart et al. found that both cold and heat extremes raise cardiopulmonary mortality, likely due to factors such as increased blood viscosity, red cell count, and vascular constriction [43]. Future research and policy must consider both temperature extremes when evaluating myocarditis burden in the context of climate change.

Several limitations should be acknowledged in this study. First, temperature exposure assessments rely on environmental data, risking ecological fallacy. Second, limited data excluded factors like socioeconomic status, climate control, infrastructure, and public health services, potentially underestimating low-environmental-temperature impacts on myocarditis deaths. Third, unmeasured confounders (e.g., occupational hazards) and methodological constraints (e.g., temporal resolution of data) could bias results, particularly in vulnerable populations.

To address these gaps, future research should:

1. Adopt multidisciplinary approaches, integrating granular environmental data with socioeconomic indices (e.g., neighborhood deprivation scores) to disentangle confounding effects
2. Implement longitudinal designs, tracking individual exposures over time to minimize ecological fallacy and capture dynamic interactions

These refinements would enhance the generalizability of findings and inform targeted public health interventions.

## Conclusion

This study robustly demonstrates that low environmental temperatures are a significant cause of mortality among individuals with myocarditis, contributing substantially to the overall disease burden. The effects extend beyond individual health, impacting healthcare systems and societal well-being.

## Supplementary Information

Supplementary material 1.

## Data Availability

All data generated or analysed during this study are included in this article. Further enquiries can be directed to the corresponding author.

## Abbreviations

GBD: Global burden of disease
DALYs: disability-adjusted life years
EAPC: Estimated annual percentage change
ASMR: age-standardized mortality rate
ASDR: age-standardized DALYs rate
95% UI: 95% uncertainty intervals
SDI: Sociodemographic Index
PAFs: population attributable fractions
CVD: cardiovascular diseases
SARS: severe acute respiratory syndrome
TBEV: Tick-borne encephalitis virus
