# Supplementary material for: Impact of low environmental temperatures on the global burden of myocarditis: insights from the 1990–2021 global burden of disease study
Source: BMC Cardiovasc Disord. 2025 Oct 10;25:731. doi: 10.1186/s12872-025-05209-2 (PMC12512437; doi:10.1186/s12872-025-05209-2)
Supplement: Supplementary file 1 — Supplementary material 1. [file 12872_2025_5209_MOESM1_ESM.docx]

**Figure Legends**

**Supplementary Figure 1.** low-temperature-related myocarditis in different GBD regions worldwide. A: number of deaths and DALYs in 2021; B: age-standardized rate of deaths and DALYs in 2021;

DALYs: disability-adjusted life years.

**
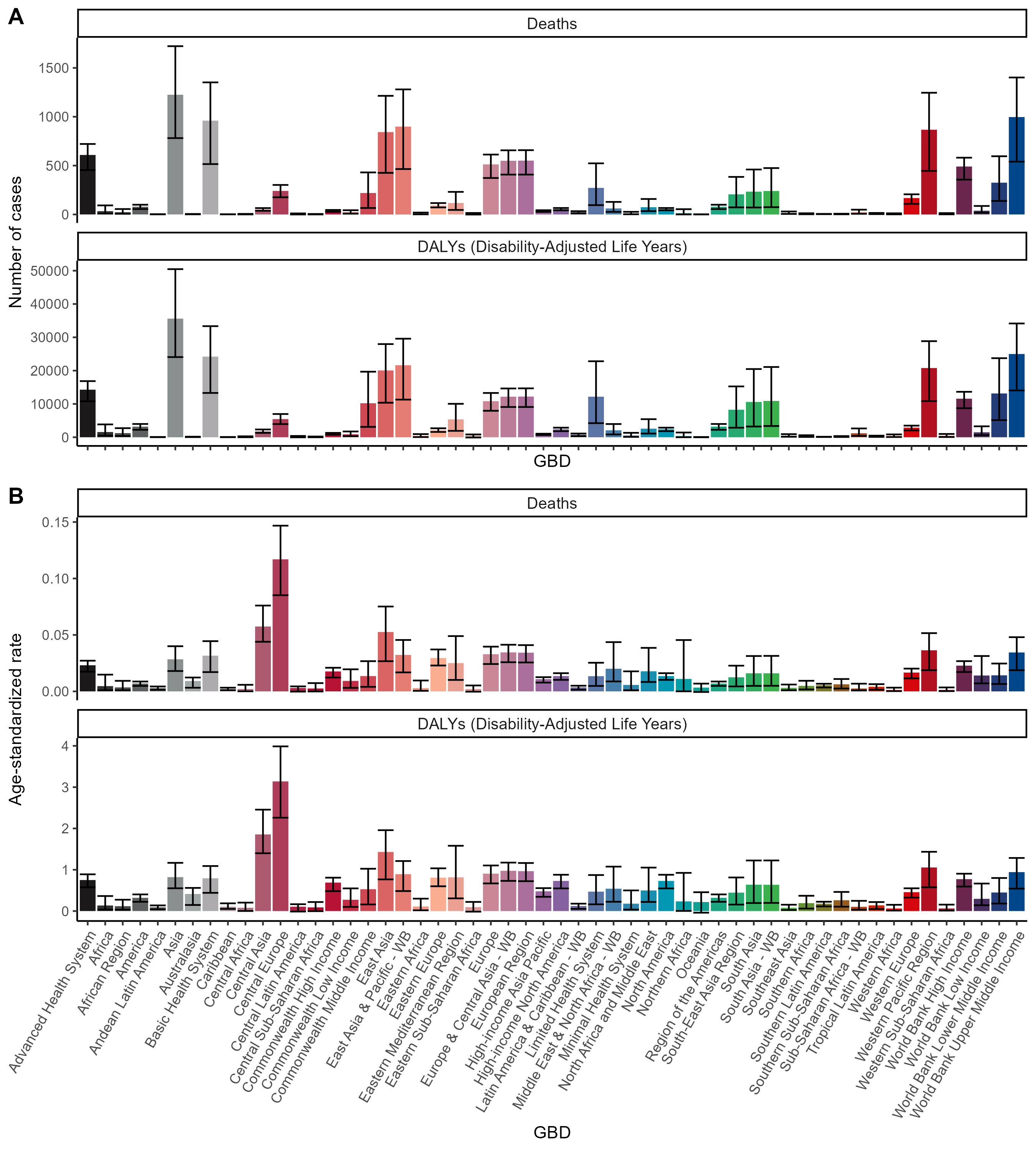
**

**Supplementary Figure 2.** The number of deaths and DALYs due to low-temperature-related myocarditis grouped by ages in 2021(A). Age-standardized deaths and DALYs per 100,000 people of low-temperature-related myocarditis grouped by ages in 2021(B).

DALYs: disability-adjusted life years.

**
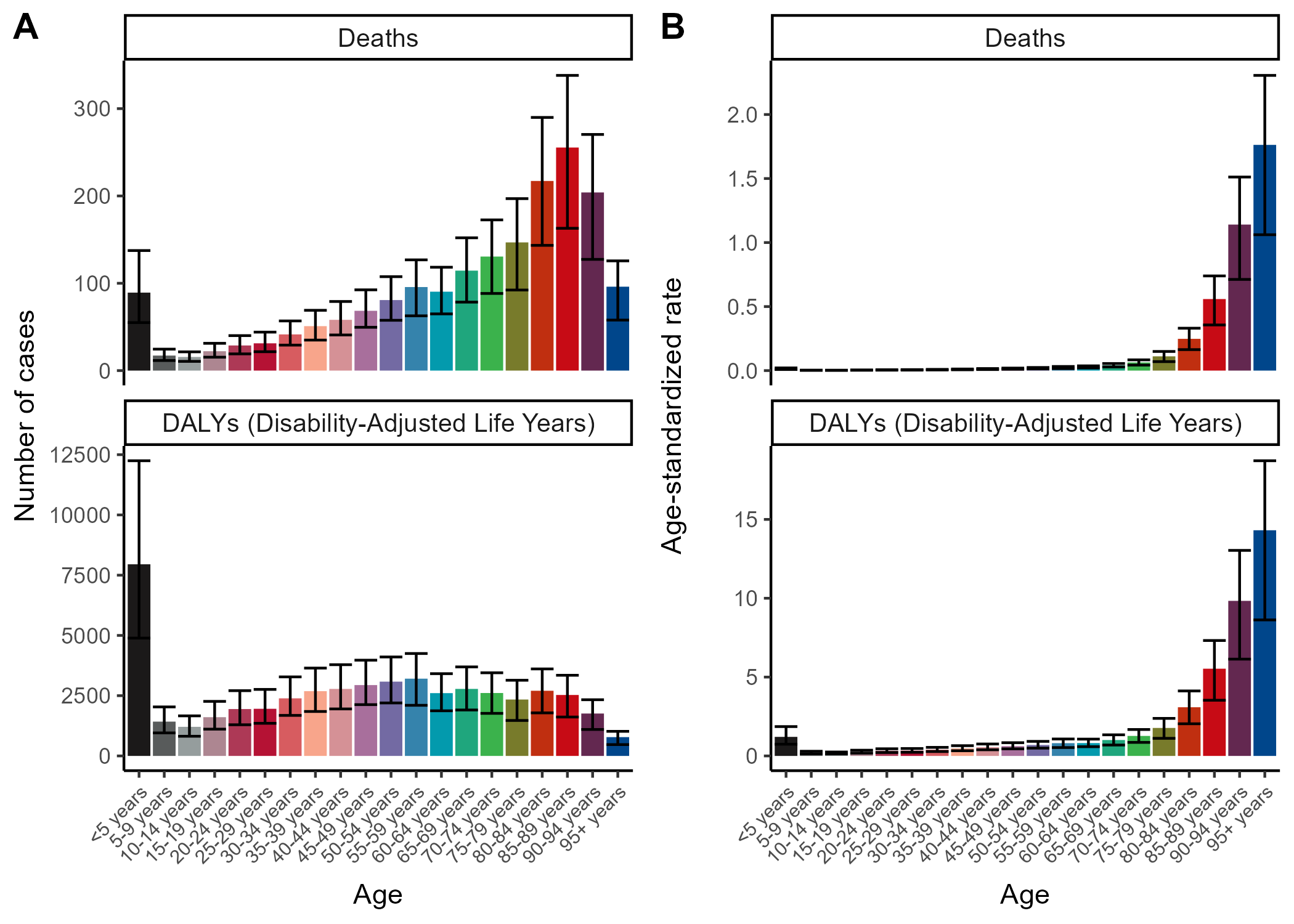
**

**Supplementary Figure 3.** The number of deaths and DALYs due to low-temperature-related myocarditis grouped by ages in 2021(A). Age-standardized mortality and DALYs per 100,000 people of low-temperature-related myocarditis grouped by ages in 2021(B).

DALYs: disability-adjusted life years.

**
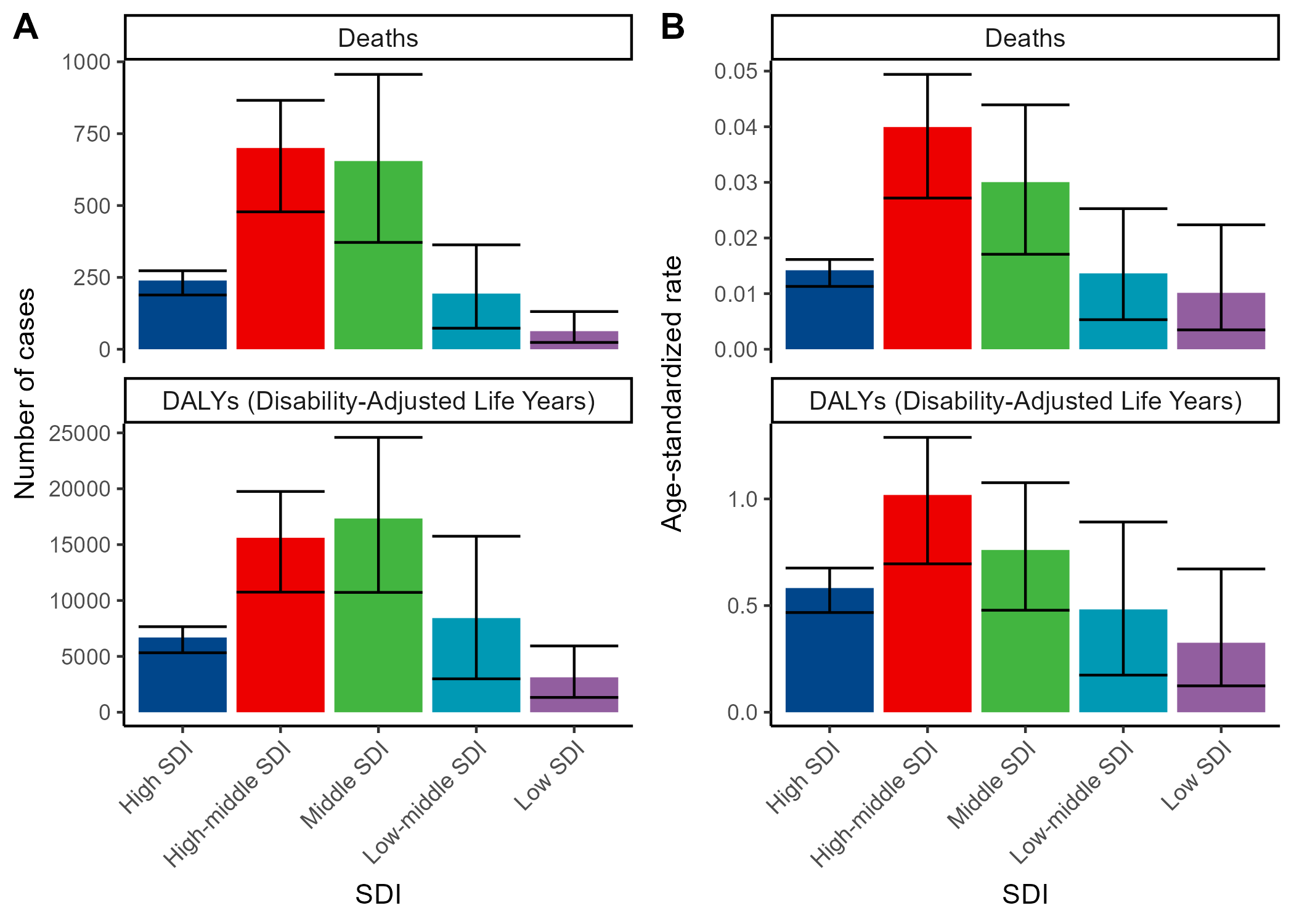
**

**Supplementary Figure 4.** The number of deaths and DALYs due to low-temperature-related myocarditis grouped by different sexes in 2021(A). Age-standardized deaths and DALYs rate per 100,000 people of low-temperature-related myocarditis grouped by different sexes in 2021(B).

DALYs: disability-adjusted life years.

**
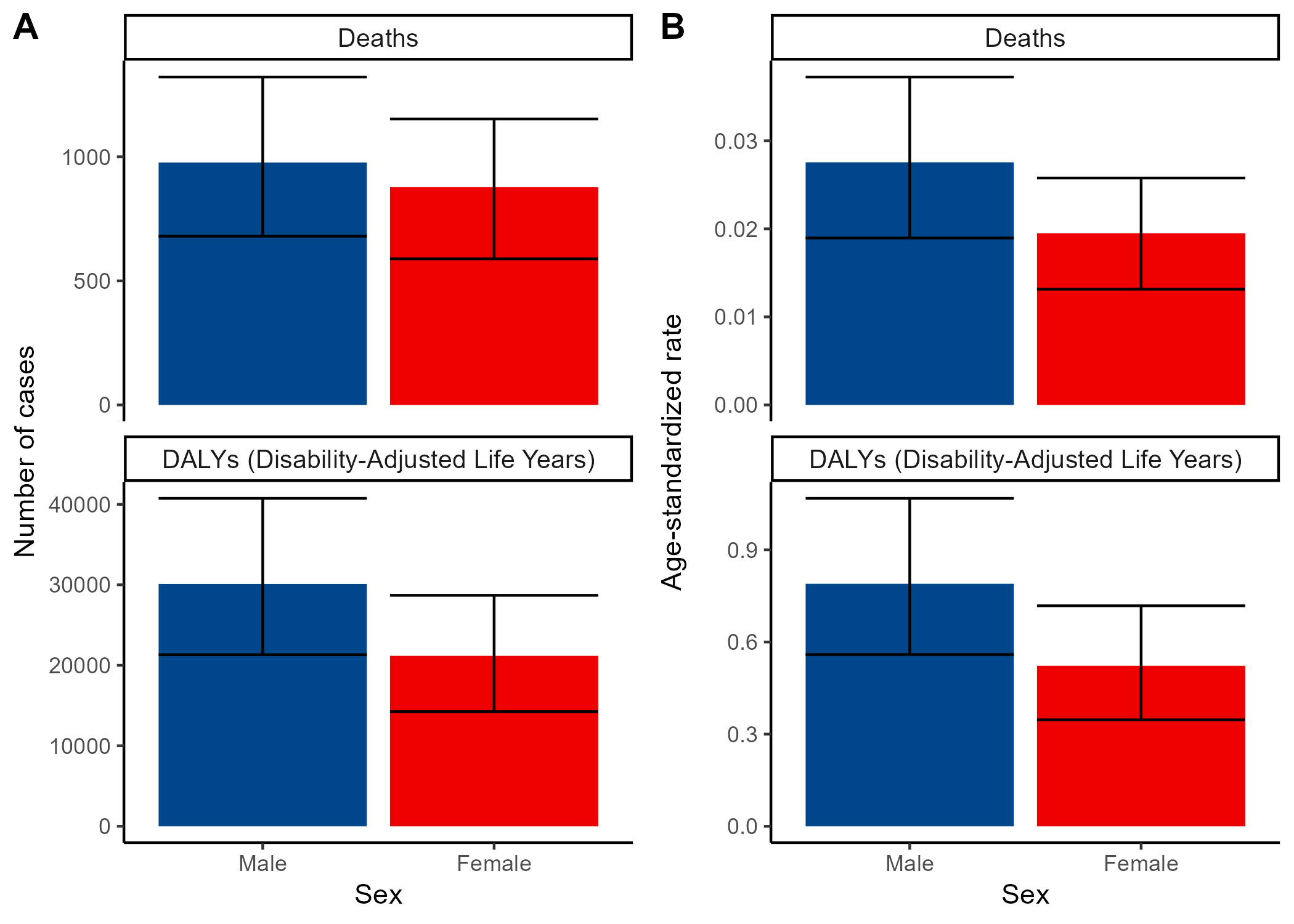
**

**Supplementary Figure 5.** Global trends in hypothermia-related myocarditis from 1990 to 2021. A: Changes in the number of deaths cases and age-standardized deaths rate from 1990-2021; B: Changes in the number of DALYs cases and age-standardized DALYs rate from 1990-2021;

DALYs: disability-adjusted life years.

**
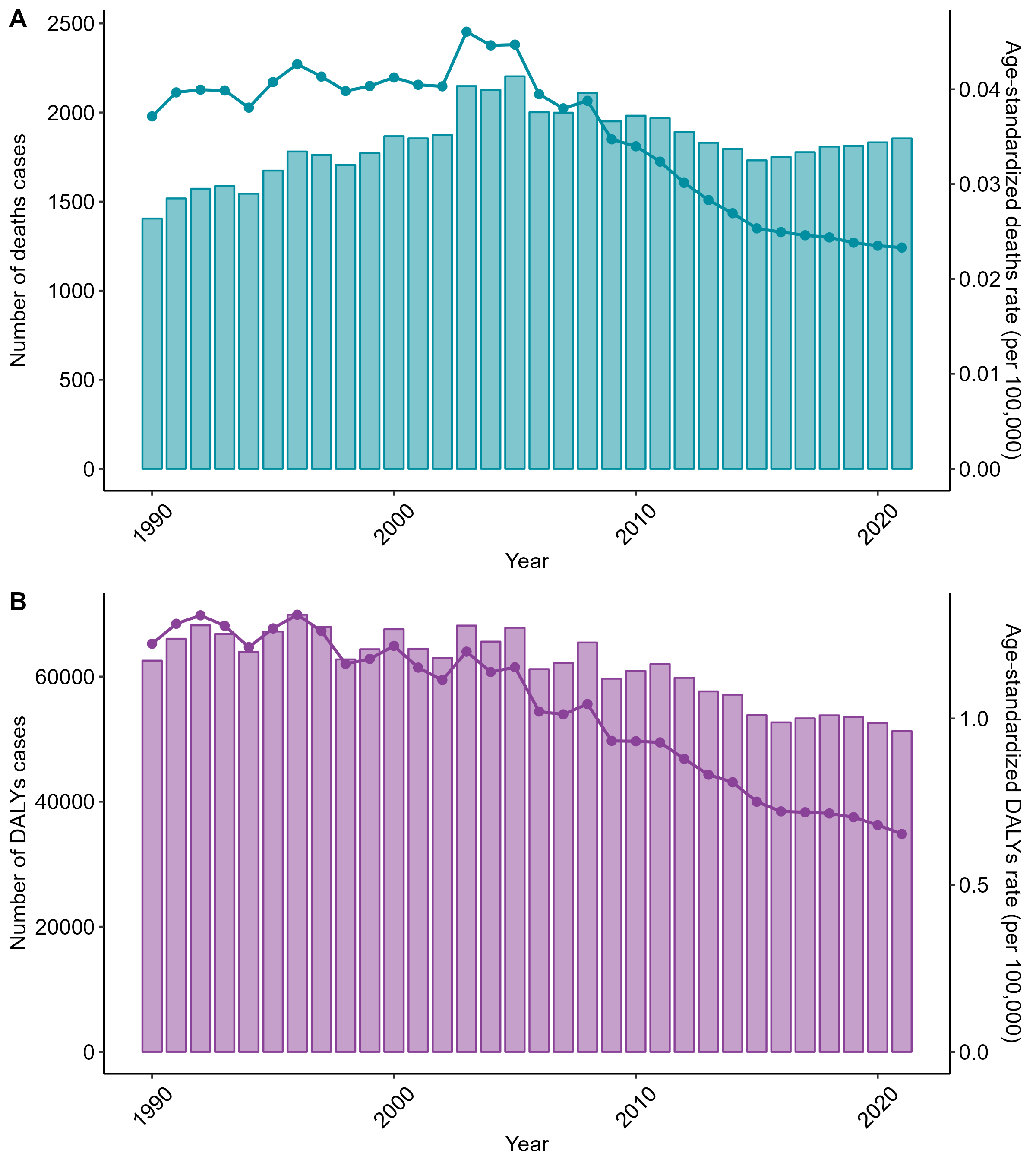
**

**Supplementary Figure 6.** The number of deaths and DALYs due to low-temperature-related myocarditis grouped by different sexes from 1990 to 2021(A). Age-standardized deaths and DALYs rate per 100,000 people of low-temperature-related myocarditis grouped by different sexes from 1990 to 2021(B).

DALYs: disability-adjusted life years.

**
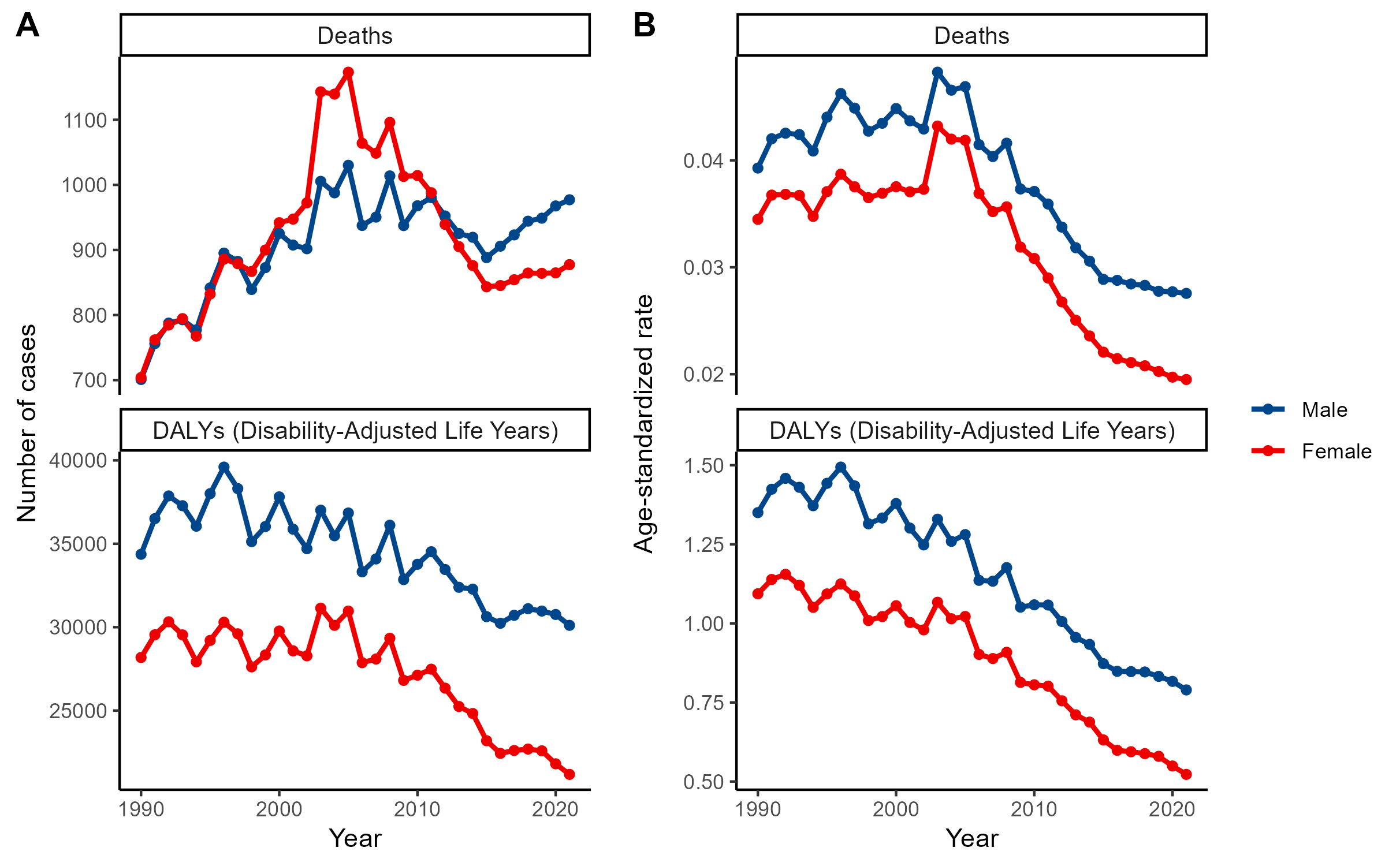
**

**Supplementary Figure 7.** The number of deaths and DALYs due to low-temperature-related myocarditis grouped by SDI from 1990 to 2021(A). Age-standardized deaths and DALYs rate per 100,000 people of low-temperature-related myocarditis grouped by SDI from 1990 to 2021(B).

DALYs: disability-adjusted life years; SDI: Socio-Demographic Index.

**
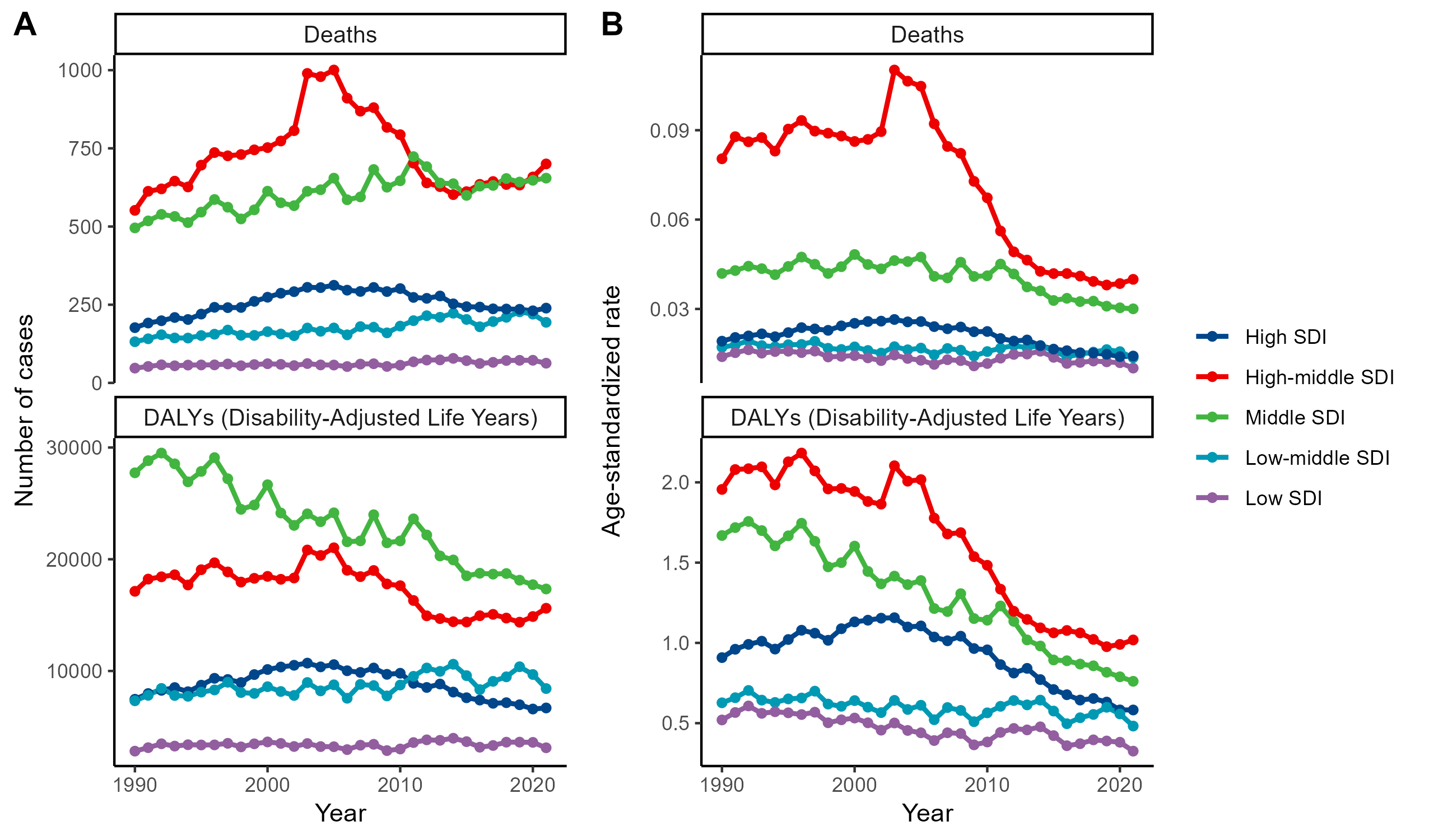
**

**Supplementary Figure 8.** The number of deaths and DALYs due to low-temperature-related myocarditis grouped by ages from 1990 to 2021(A). Age-standardized mortality and DALYs per 100,000 people of low-temperature-related myocarditis grouped by ages from 1990 to 2021(B).

DALYs: disability-adjusted life years.


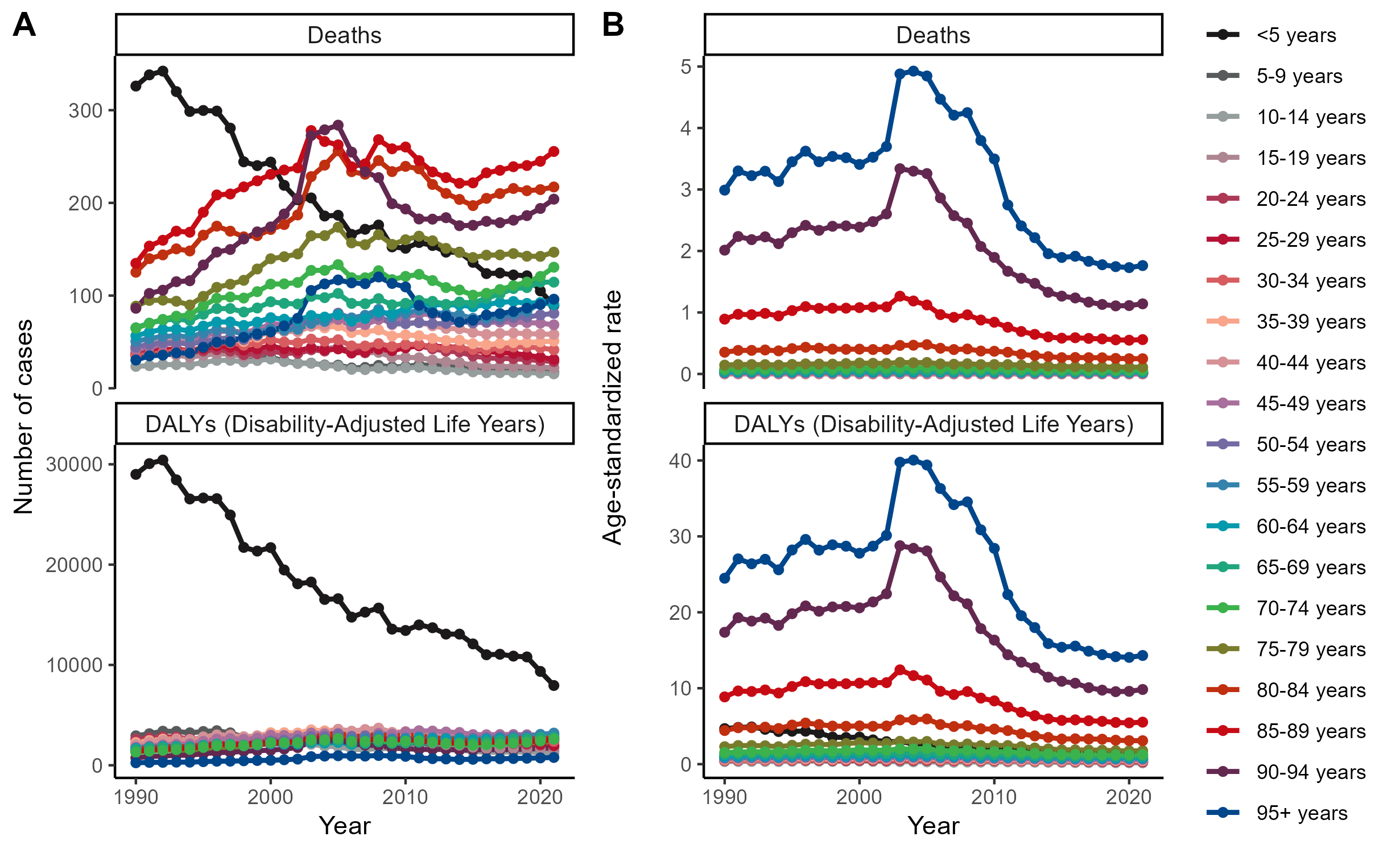


.

**Supplementary Table 1. Deaths cases and age-standardized rate of Low-temperature-related myocarditis and their EAPCs from 1990 to 2021 at the countries and territories levels**

|  | **No.1990(95%UI)** | **ASMR.1990(95%UI)** | **No.2021(95%UI)** | **ASMR.2021(95%UI)** | **EAPC(%)(95%UI)** |
| --- | --- | --- | --- | --- | --- |
| **Advanced Health System** | 526 (380-631) | 0.04 (0.03-0.05) | 609 (454-721) | 0.02 (0.02-0.03) | -2.53 (-3.21--1.84) |
| **Africa** | 40 (11-89) | 0.01 (0-0.02) | 34 (9-93) | 0 (0-0.01) | -2.76 (-3.01--2.5) |
| **African Region** | 30 (8-56) | 0.01 (0-0.02) | 24 (7-55) | 0 (0-0.01) | -3.14 (-3.4--2.89) |
| **America** | 73 (53-91) | 0.01 (0.01-0.01) | 80 (55-99) | 0.01 (0-0.01) | -1.71 (-2.17--1.25) |
| **Andean Latin America** | 3 (1-5) | 0.01 (0-0.02) | 2 (1-3) | 0 (0-0) | -5.09 (-5.41--4.78) |
| **Asia** | 850 (585-1229) | 0.04 (0.03-0.05) | 1225 (782-1722) | 0.03 (0.02-0.04) | -0.97 (-1.27--0.67) |
| **Australasia** | 5 (0-7) | 0.02 (0-0.04) | 4 (1-5) | 0.01 (0-0.01) | -3.74 (-4.37--3.1) |
| **Basic Health System** | 683 (499-943) | 0.04 (0.03-0.06) | 960 (515-1352) | 0.03 (0.02-0.04) | -1.08 (-1.45--0.71) |
| **Caribbean** | 1 (0-2) | 0 (0-0) | 1 (1-2) | 0 (0-0) | -1.43 (-2.16--0.7) |
| **Central Africa** | 3 (0-6) | 0.01 (0-0.01) | 2 (0-6) | 0 (0-0.01) | -3.94 (-4.46--3.42) |
| **Central Asia** | 18 (13-23) | 0.03 (0.03-0.04) | 49 (37-65) | 0.06 (0.04-0.08) | 2.1 (1.43-2.76) |
| **Central Europe** | 108 (83-141) | 0.09 (0.07-0.11) | 241 (176-302) | 0.12 (0.09-0.15) | -0.55 (-1.12-0.01) |
| **Central Latin America** | 2 (1-3) | 0 (0-0) | 7 (0-10) | 0 (0-0) | 1.92 (1.4-2.43) |
| **Central Sub-Saharan Africa** | 2 (0-5) | 0.01 (0-0.01) | 2 (0-5) | 0 (0-0.01) | -3.23 (-3.77--2.69) |
| **Commonwealth High Income** | 38 (26-45) | 0.03 (0.02-0.04) | 38 (27-46) | 0.02 (0.01-0.02) | -2.31 (-2.79--1.84) |
| **Commonwealth Low Income** | 15 (4-31) | 0.01 (0-0.03) | 22 (8-43) | 0.01 (0-0.02) | -0.66 (-1.12--0.21) |
| **Commonwealth Middle Income** | 146 (44-301) | 0.02 (0.01-0.04) | 219 (66-430) | 0.01 (0-0.03) | -0.62 (-0.94--0.3) |
| **East Asia** | 594 (422-834) | 0.08 (0.05-0.11) | 843 (426-1214) | 0.05 (0.03-0.08) | -1.32 (-1.76--0.88) |
| **East Asia & Pacific - WB** | 638 (459-884) | 0.05 (0.03-0.07) | 899 (465-1280) | 0.03 (0.02-0.05) | -1.72 (-2.32--1.11) |
| **Eastern Africa** | 11 (0-22) | 0.01 (0-0.02) | 7 (1-19) | 0 (0-0.01) | -3.8 (-4.22--3.37) |
| **Eastern Europe** | 64 (43-77) | 0.03 (0.02-0.04) | 93 (72-116) | 0.03 (0.02-0.04) | -0.59 (-1.06--0.13) |
| **Eastern Mediterranean Region** | 97 (31-197) | 0.04 (0.01-0.09) | 118 (46-231) | 0.03 (0.01-0.05) | -1.47 (-1.74--1.19) |
| **Eastern Sub-Saharan Africa** | 10 (-4-22) | 0.01 (0-0.01) | 7 (-1-15) | 0 (0-0.01) | -3.55 (-3.87--3.22) |
| **Europe** | 440 (309-529) | 0.05 (0.04-0.06) | 513 (373-613) | 0.03 (0.02-0.04) | -2.57 (-3.38--1.76) |
| **Europe & Central Asia - WB** | 447 (316-538) | 0.05 (0.04-0.06) | 549 (408-656) | 0.03 (0.03-0.04) | -2.58 (-3.52--1.64) |
| **European Region** | 448 (316-539) | 0.05 (0.04-0.06) | 551 (408-658) | 0.03 (0.03-0.04) | -2.27 (-3.06--1.48) |
| **High-income Asia Pacific** | 26 (19-33) | 0.02 (0.01-0.02) | 36 (26-43) | 0.01 (0.01-0.01) | -1.92 (-2.39--1.46) |
| **High-income North America** | 56 (40-67) | 0.02 (0.01-0.02) | 56 (42-66) | 0.01 (0.01-0.02) | -1.37 (-1.86--0.89) |
| **Latin America & Caribbean - WB** | 17 (11-24) | 0.01 (0-0.01) | 24 (10-33) | 0 (0-0.01) | -1.88 (-2.42--1.33) |
| **Limited Health System** | 183 (62-358) | 0.02 (0.01-0.04) | 272 (96-523) | 0.01 (0-0.03) | -0.68 (-0.97--0.4) |
| **Middle East & North Africa - WB** | 61 (22-116) | 0.04 (0.01-0.09) | 62 (26-128) | 0.02 (0.01-0.04) | -2.21 (-2.53--1.88) |
| **Minimal Health System** | 10 (2-26) | 0.01 (0-0.04) | 10 (2-27) | 0.01 (0-0.02) | -2.74 (-2.98--2.5) |
| **North Africa and Middle East** | 71 (24-148) | 0.04 (0.01-0.08) | 74 (34-158) | 0.02 (0.01-0.04) | -2.18 (-2.46--1.9) |
| **North America** | 56 (40-67) | 0.02 (0.01-0.02) | 56 (42-66) | 0.01 (0.01-0.02) | -1.37 (-1.86--0.89) |
| **Northern Africa** | 12 (2-39) | 0.02 (0-0.06) | 13 (0-54) | 0.01 (0-0.05) | -1.56 (-1.9--1.21) |
| **Oceania** | 0 (0-1) | 0 (0-0.01) | 1 (0-1) | 0 (0-0.01) | -0.76 (-1.1--0.42) |
| **Region of the Americas** | 73 (53-91) | 0.01 (0.01-0.01) | 80 (55-99) | 0.01 (0-0.01) | -1.71 (-2.17--1.25) |
| **South-East Asia Region** | 134 (42-266) | 0.02 (0.01-0.03) | 206 (72-384) | 0.01 (0-0.02) | -0.59 (-0.89--0.27) |
| **South Asia** | 148 (39-314) | 0.02 (0-0.04) | 233 (71-460) | 0.02 (0-0.03) | -0.32 (-0.65-0.01) |
| **South Asia - WB** | 155 (41-330) | 0.02 (0.01-0.04) | 240 (74-475) | 0.02 (0-0.03) | -0.48 (-0.81--0.16) |
| **Southeast Asia** | 14 (5-28) | 0.01 (0-0.01) | 15 (5-30) | 0 (0-0.01) | -3.07 (-3.63--2.52) |
| **Southern Africa** | 7 (1-11) | 0.01 (0-0.02) | 6 (2-12) | 0 (0-0.01) | -3.02 (-3.38--2.66) |
| **Southern Latin America** | 6 (4-8) | 0.01 (0.01-0.02) | 4 (3-6) | 0.01 (0-0.01) | -3.07 (-3.73--2.4) |
| **Southern Sub-Saharan Africa** | 5 (2-7) | 0.01 (0.01-0.02) | 4 (2-7) | 0.01 (0-0.01) | -3.08 (-3.47--2.69) |
| **Sub-Saharan Africa - WB** | 28 (7-55) | 0.01 (0-0.01) | 20 (6-49) | 0 (0-0.01) | -4.33 (-4.84--3.82) |
| **Tropical Latin America** | 5 (3-8) | 0 (0-0.01) | 10 (3-15) | 0 (0-0.01) | -1.67 (-2.66--0.67) |
| **Western Africa** | 8 (2-19) | 0.01 (0-0.01) | 5 (1-12) | 0 (0-0) | -5.43 (-6.07--4.78) |
| **Western Europe** | 258 (174-321) | 0.05 (0.03-0.06) | 168 (109-206) | 0.02 (0.01-0.02) | -4.53 (-5.76--3.28) |
| **Western Pacific Region** | 620 (447-860) | 0.05 (0.04-0.08) | 867 (446-1246) | 0.04 (0.02-0.05) | -1.51 (-1.89--1.14) |
| **Western Sub-Saharan Africa** | 9 (2-21) | 0.01 (0-0.01) | 5 (1-14) | 0 (0-0) | -5.34 (-5.98--4.7) |
| **World Bank High Income** | 446 (314-539) | 0.04 (0.03-0.05) | 490 (356-581) | 0.02 (0.02-0.03) | -2.77 (-3.5--2.04) |
| **World Bank Low Income** | 34 (14-70) | 0.02 (0.01-0.04) | 38 (18-87) | 0.01 (0.01-0.03) | -1.44 (-1.66--1.22) |
| **World Bank Lower Middle Income** | 218 (81-405) | 0.02 (0.01-0.03) | 325 (138-596) | 0.01 (0.01-0.02) | -0.73 (-0.98--0.49) |
| **World Bank Upper Middle Income** | 704 (516-953) | 0.05 (0.04-0.06) | 997 (541-1402) | 0.03 (0.02-0.05) | -1.29 (-1.64--0.93) |
| **Country** |  |  |  |  |  |
| **Afghanistan** | 5 (1-16) | 0.06 (0.01-0.23) | 6 (1-19) | 0.05 (0.01-0.18) | -0.86 (-1.06--0.66) |
| **Albania** | 2 (1-3) | 0.08 (0.04-0.14) | 2 (1-3) | 0.05 (0.02-0.09) | -2.01 (-2.33--1.69) |
| **Algeria** | 4 (1-14) | 0.04 (0-0.12) | 6 (1-22) | 0.02 (0-0.08) | -1.55 (-1.93--1.18) |
| **American Samoa** | 0 (0-0) | 0 (0-0) | 0 (0-0) | 0 (0-0) | -1.42 (-2.57--0.24) |
| **Andorra** | 0 (0-0) | 0.04 (0.02-0.07) | 0 (0-0) | 0.02 (0.01-0.03) | -3.09 (-3.31--2.87) |
| **Angola** | 1 (0-1) | 0.01 (0-0.02) | 1 (0-1) | 0 (0-0.01) | -2.95 (-3.55--2.34) |
| **Antigua and Barbuda** | 0 (0-0) | 0.01 (0-0.01) | 0 (0-0) | 0 (0-0) | -1.51 (-2.78--0.22) |
| **Argentina** | 5 (3-7) | 0.02 (0.01-0.02) | 3 (2-4) | 0.01 (0-0.01) | -3.21 (-3.85--2.58) |
| **Armenia** | 0 (0-0) | 0.01 (0.01-0.02) | 0 (0-0) | 0.01 (0-0.01) | -3.17 (-4.29--2.04) |
| **Australia** | 4 (0-6) | 0.02 (0-0.04) | 2 (1-3) | 0.01 (0-0.01) | -4.61 (-5.24--3.97) |
| **Austria** | 0 (0-0) | 0 (0-0.01) | 3 (2-3) | 0.01 (0.01-0.02) | 4.2 (1.91-6.53) |
| **Azerbaijan** | 4 (2-7) | 0.08 (0.04-0.14) | 7 (3-14) | 0.06 (0.03-0.13) | -1.85 (-2.28--1.42) |
| **Bahamas** | 0 (0-0) | 0 (0-0) | 0 (0-0) | 0 (0-0) | -0.97 (-2.44-0.52) |
| **Bahrain** | 0 (0-0) | 0.02 (0-0.03) | 0 (0-0) | 0.01 (0-0.01) | -3.65 (-4.09--3.19) |
| **Bangladesh** | 11 (3-25) | 0.02 (0-0.05) | 19 (6-40) | 0.01 (0-0.03) | -0.31 (-0.83-0.21) |
| **Barbados** | 0 (0-0) | 0 (0-0) | 0 (0-0) | 0 (0-0) | -1.31 (-3.16-0.59) |
| **Belarus** | 3 (2-5) | 0.03 (0.02-0.04) | 1 (1-2) | 0.01 (0.01-0.01) | -4.54 (-5.16--3.92) |
| **Belgium** | 1 (0-1) | 0.01 (0-0.01) | 4 (3-5) | 0.01 (0.01-0.02) | 3.21 (1.19-5.26) |
| **Belize** | 0 (0-0) | 0 (0-0.01) | 0 (0-0) | 0 (0-0) | -4.06 (-5.08--3.04) |
| **Benin** | 0 (0-0) | 0 (0-0.01) | 0 (0-0) | 0 (0-0) | -6.2 (-6.9--5.5) |
| **Bermuda** | 0 (0-0) | 0 (0-0.01) | 0 (0-0) | 0 (0-0.01) | -0.95 (-1.8--0.08) |
| **Bhutan** | 0 (0-0) | 0.03 (0.01-0.06) | 0 (0-0) | 0.02 (0.01-0.04) | -1.48 (-1.69--1.27) |
| **Bolivia (Plurinational State of)** | 1 (0-1) | 0.02 (0.01-0.03) | 1 (0-1) | 0.01 (0-0.01) | -2.88 (-3.08--2.68) |
| **Bosnia and Herzegovina** | 3 (2-6) | 0.09 (0.04-0.19) | 4 (2-7) | 0.06 (0.03-0.12) | -2.1 (-2.42--1.79) |
| **Botswana** | 0 (0-0) | 0.01 (0-0.02) | 0 (0-0) | 0.01 (0-0.01) | -2.68 (-3.2--2.16) |
| **Brazil** | 5 (2-7) | 0 (0-0.01) | 10 (3-15) | 0 (0-0.01) | -1.65 (-2.66--0.63) |
| **Brunei Darussalam** | 0 (0-0) | 0 (0-0.01) | 0 (0-0) | 0 (0-0) | -4.43 (-5.55--3.3) |
| **Bulgaria** | 6 (4-7) | 0.07 (0.05-0.09) | 14 (9-19) | 0.12 (0.08-0.16) | -0.27 (-1.2-0.66) |
| **Burkina Faso** | 0 (0-1) | 0.01 (0-0.02) | 0 (0-1) | 0 (0-0) | -5.42 (-6.46--4.37) |
| **Burundi** | 0 (-1-1) | 0.01 (-0.01-0.01) | 0 (0-0) | 0 (0-0.01) | -4.5 (-5.18--3.81) |
| **Cabo Verde** | 0 (0-0) | 0 (0-0) | 0 (0-0) | 0 (0-0) | -2.6 (-4.03--1.16) |
| **Cambodia** | 0 (0-1) | 0 (0-0.01) | 0 (0-1) | 0 (0-0.01) | -4.05 (-5.46--2.61) |
| **Cameroon** | 0 (0-1) | 0.01 (0-0.01) | 0 (0-1) | 0 (0-0) | -5.03 (-5.68--4.36) |
| **Canada** | 2 (2-2) | 0.01 (0.01-0.01) | 4 (4-5) | 0.01 (0.01-0.01) | 1.17 (0.28-2.07) |
| **Central African Republic** | 0 (0-0) | 0 (0-0.01) | 0 (0-0) | 0 (0-0) | -3.64 (-4.36--2.91) |
| **Chad** | 0 (0-1) | 0.01 (0-0.02) | 0 (0-1) | 0 (0-0.01) | -4.68 (-5.55--3.81) |
| **Chile** | 1 (1-1) | 0.01 (0.01-0.01) | 1 (0-1) | 0 (0-0.01) | -2.16 (-3.08--1.24) |
| **China** | 582 (414-820) | 0.08 (0.05-0.11) | 818 (409-1186) | 0.05 (0.03-0.08) | -1.32 (-1.78--0.87) |
| **Colombia** | 1 (0-1) | 0 (0-0) | 2 (-1-3) | 0 (0-0.01) | -0.49 (-1.25-0.27) |
| **Comoros** | 0 (0-0) | 0 (0-0.01) | 0 (0-0) | 0 (0-0) | -3.75 (-4.23--3.26) |
| **Congo** | 0 (0-0) | 0.01 (0-0.01) | 0 (0-0) | 0 (0-0) | -4.28 (-4.86--3.7) |
| **Cook Islands** | 0 (0-0) | 0 (0-0) | 0 (0-0) | 0 (0-0) | -6.32 (-10--2.49) |
| **Costa Rica** | 0 (0-0) | 0 (0-0.01) | 0 (0-0) | 0 (0-0.01) | -3.02 (-4.09--1.94) |
| **Croatia** | 6 (4-7) | 0.12 (0.08-0.14) | 13 (9-17) | 0.15 (0.1-0.2) | -2.55 (-4.15--0.91) |
| **Cuba** | 0 (0-0) | 0 (0-0) | 0 (0-0) | 0 (0-0) | -1.72 (-2.65--0.78) |
| **Cyprus** | 0 (0-0) | 0.02 (0-0.04) | 0 (0-0) | 0 (0-0.01) | -6.21 (-6.78--5.65) |
| **Czechia** | 2 (2-3) | 0.02 (0.01-0.02) | 5 (4-7) | 0.03 (0.02-0.03) | 0.3 (-0.14-0.75) |
| **Cote d'Ivoire** | 0 (0-1) | 0 (0-0.01) | 0 (0-0) | 0 (0-0) | -5.64 (-6.36--4.91) |
| **Democratic People's Republic of Korea** | 11 (7-16) | 0.09 (0.05-0.14) | 19 (11-36) | 0.08 (0.05-0.16) | -0.11 (-0.39-0.16) |
| **Democratic Republic of the Congo** | 2 (0-3) | 0.01 (0-0.01) | 1 (0-3) | 0 (0-0.01) | -3.24 (-3.8--2.67) |
| **Denmark** | 1 (0-1) | 0.01 (0.01-0.01) | 2 (1-2) | 0.01 (0.01-0.02) | 1.93 (0.09-3.8) |
| **Djibouti** | 0 (0-0) | 0.01 (0-0.02) | 0 (0-0) | 0 (0-0.01) | -2.99 (-3.65--2.32) |
| **Dominica** | 0 (0-0) | 0.01 (0-0.01) | 0 (0-0) | 0 (0-0.01) | -2.47 (-3.72--1.19) |
| **Dominican Republic** | 0 (0-0) | 0 (0-0) | 0 (0-0) | 0 (0-0) | -1.2 (-1.94--0.45) |
| **Ecuador** | 1 (0-1) | 0.01 (0.01-0.01) | 0 (0-1) | 0 (0-0) | -6.06 (-6.72--5.39) |
| **Egypt** | 1 (0-2) | 0 (0-0) | 0 (0-1) | 0 (0-0) | -4.42 (-4.86--3.97) |
| **El Salvador** | 0 (0-0) | 0 (0-0) | 0 (0-0) | 0 (0-0) | -3.74 (-5.25--2.21) |
| **Equatorial Guinea** | 0 (0-0) | 0.01 (0-0.01) | 0 (0-0) | 0 (0-0) | -7.95 (-8.48--7.42) |
| **Eritrea** | 0 (0-0) | 0.01 (0-0.01) | 0 (0-0) | 0 (0-0.01) | -3.46 (-4.05--2.87) |
| **Estonia** | 0 (0-0) | 0.01 (0-0.01) | 0 (0-0) | 0 (0-0.01) | -2.81 (-3.41--2.2) |
| **Eswatini** | 0 (0-0) | 0.01 (0.01-0.02) | 0 (0-0) | 0.01 (0-0.01) | -2.91 (-3.34--2.48) |
| **Ethiopia** | 4 (0-9) | 0.01 (0-0.02) | 2 (0-4) | 0 (0-0.01) | -4.48 (-4.95--4) |
| **Fiji** | 0 (0-0) | 0 (0-0) | 0 (0-0) | 0 (0-0) | -2.57 (-3.87--1.26) |
| **Finland** | 1 (0-2) | 0.02 (0.01-0.03) | 2 (1-3) | 0.02 (0.01-0.02) | 0.25 (-1.23-1.75) |
| **France** | 1 (1-1) | 0 (0-0) | 10 (7-12) | 0.01 (0-0.01) | 4.27 (2.22-6.37) |
| **Gabon** | 0 (0-0) | 0.01 (0-0.01) | 0 (0-0) | 0 (0-0) | -5.86 (-6.38--5.34) |
| **Gambia** | 0 (0-0) | 0 (0-0.01) | 0 (0-0) | 0 (0-0) | -3.25 (-4.04--2.46) |
| **Georgia** | 4 (2-6) | 0.07 (0.04-0.1) | 3 (2-4) | 0.05 (0.03-0.08) | -1.81 (-2.49--1.12) |
| **Germany** | 15 (10-19) | 0.01 (0.01-0.02) | 28 (22-34) | 0.02 (0.01-0.02) | 0.38 (-0.71-1.48) |
| **Ghana** | 1 (0-1) | 0.01 (0-0.01) | 0 (0-0) | 0 (0-0) | -7.01 (-7.77--6.24) |
| **Greece** | 0 (0-0) | 0 (0-0) | 1 (1-1) | 0 (0-0.01) | 2.48 (0.22-4.79) |
| **Greenland** | 0 (0-0) | 0.01 (-0.01-0.03) | 0 (0-0) | 0.01 (0-0.02) | -0.46 (-0.89--0.02) |
| **Grenada** | 0 (0-0) | 0 (0-0) | 0 (0-0) | 0 (0-0) | -3.08 (-4.97--1.14) |
| **Guam** | 0 (0-0) | 0 (0-0) | 0 (0-0) | 0 (0-0) | -3.88 (-5.6--2.14) |
| **Guatemala** | 0 (0-0) | 0 (0-0.01) | 0 (0-1) | 0 (0-0) | -1.95 (-2.5--1.39) |
| **Guinea** | 0 (0-1) | 0.01 (0-0.01) | 0 (0-0) | 0 (0-0) | -4.19 (-4.72--3.66) |
| **Guinea-Bissau** | 0 (0-0) | 0 (0-0.01) | 0 (0-0) | 0 (0-0) | -3.46 (-4.88--2.02) |
| **Guyana** | 0 (0-0) | 0.01 (0-0.01) | 0 (0-0) | 0.01 (0-0.02) | 0.75 (-0.72-2.25) |
| **Haiti** | 0 (0-1) | 0.01 (0-0.01) | 0 (0-1) | 0 (0-0.01) | -2.53 (-4.3--0.72) |
| **Honduras** | 0 (0-0) | 0 (0-0.01) | 0 (0-0) | 0 (0-0) | -1.71 (-4.41-1.07) |
| **Hungary** | 3 (3-4) | 0.03 (0.02-0.04) | 4 (3-6) | 0.02 (0.02-0.03) | -1.26 (-1.8--0.72) |
| **Iceland** | 0 (0-0) | 0 (0-0) | 0 (0-0) | 0.01 (0.01-0.02) | 3.73 (1.63-5.86) |
| **India** | 100 (25-213) | 0.02 (0-0.04) | 157 (45-309) | 0.01 (0-0.03) | -0.33 (-0.68-0.03) |
| **Indonesia** | 3 (0-6) | 0 (0-0.01) | 2 (-3-5) | 0 (0-0) | -3.02 (-4.31--1.7) |
| **Iran (Islamic Republic of)** | 11 (5-18) | 0.05 (0.02-0.08) | 13 (6-20) | 0.02 (0.01-0.03) | -1.95 (-2.6--1.29) |
| **Iraq** | 26 (8-46) | 0.21 (0.06-0.42) | 24 (8-44) | 0.1 (0.03-0.21) | -2.22 (-2.59--1.84) |
| **Ireland** | 0 (0-0) | 0.01 (0.01-0.01) | 3 (2-3) | 0.03 (0.03-0.04) | 5.13 (3.15-7.14) |
| **Israel** | 0 (0-0) | 0 (0-0) | 0 (0-1) | 0 (0-0) | 1 (-1.24-3.29) |
| **Italy** | 199 (128-252) | 0.28 (0.18-0.35) | 57 (30-74) | 0.03 (0.01-0.03) | -8.77 (-10.42--7.09) |
| **Jamaica** | 0 (0-0) | 0 (0-0) | 0 (0-0) | 0 (0-0) | -1.24 (-1.99--0.49) |
| **Japan** | 15 (11-18) | 0.01 (0.01-0.01) | 29 (20-35) | 0.01 (0.01-0.01) | -1.05 (-1.8--0.3) |
| **Jordan** | 0 (0-0) | 0.01 (0-0.02) | 0 (0-0) | 0 (0-0) | -3.87 (-4.36--3.37) |
| **Kazakhstan** | 1 (1-1) | 0.01 (0-0.01) | 28 (20-40) | 0.16 (0.11-0.22) | 13.61 (11.45-15.81) |
| **Kenya** | 0 (-1-1) | 0 (0-0.01) | 0 (0-1) | 0 (0-0) | -2.73 (-3.19--2.28) |
| **Kiribati** | 0 (0-0) | 0 (0-0) | 0 (0-0) | 0 (0-0) | -0.2 (-4.83-4.65) |
| **Kuwait** | 0 (0-1) | 0.04 (0.01-0.07) | 1 (0-1) | 0.02 (0.01-0.05) | -1.66 (-2.49--0.82) |
| **Kyrgyzstan** | 1 (1-1) | 0.02 (0.02-0.03) | 2 (1-2) | 0.04 (0.03-0.05) | 2.4 (1.39-3.43) |
| **Lao People's Democratic Republic** | 0 (0-1) | 0.01 (0-0.03) | 0 (0-1) | 0.01 (0-0.02) | -2.04 (-2.77--1.31) |
| **Latvia** | 0 (0-0) | 0.01 (0-0.01) | 0 (0-0) | 0.01 (0-0.01) | -0.57 (-1.03--0.1) |
| **Lebanon** | 1 (0-1) | 0.04 (0.01-0.07) | 1 (0-2) | 0.01 (0-0.02) | -3.23 (-3.62--2.84) |
| **Lesotho** | 0 (0-0) | 0.03 (0.01-0.05) | 0 (0-0) | 0.02 (0.01-0.03) | -2.25 (-2.5--2) |
| **Liberia** | 0 (0-0) | 0 (0-0.01) | 0 (0-0) | 0 (0-0) | -4.85 (-5.48--4.22) |
| **Libya** | 1 (0-3) | 0.02 (0-0.08) | 1 (0-2) | 0.01 (0-0.05) | -1.97 (-2.4--1.53) |
| **Lithuania** | 0 (0-0) | 0.01 (0-0.01) | 0 (0-0) | 0.01 (0-0.01) | -1.01 (-1.66--0.37) |
| **Luxembourg** | 0 (0-0) | 0.01 (0.01-0.01) | 0 (0-0) | 0.02 (0.01-0.02) | 2.92 (1.76-4.1) |
| **Madagascar** | 1 (0-2) | 0.01 (0-0.02) | 1 (0-2) | 0 (0-0.01) | -2.88 (-3.17--2.6) |
| **Malawi** | 0 (0-1) | 0 (0-0.01) | 0 (0-1) | 0 (0-0.01) | -2.11 (-2.56--1.65) |
| **Malaysia** | 0 (0-0) | 0 (0-0) | 0 (0-0) | 0 (0-0) | -4.55 (-5.5--3.58) |
| **Maldives** | 0 (0-0) | 0 (0-0) | 0 (0-0) | 0 (0-0) | -4.24 (-5.06--3.42) |
| **Mali** | 0 (0-1) | 0.01 (0-0.02) | 0 (0-0) | 0 (0-0) | -4.66 (-5.82--3.48) |
| **Malta** | 0 (0-0) | 0.01 (-0.01-0.02) | 0 (0-0) | 0.02 (-0.01-0.04) | 1.49 (-0.68-3.7) |
| **Marshall Islands** | 0 (0-0) | 0 (0-0) | 0 (0-0) | 0 (0-0) | -8.04 (-9.42--6.64) |
| **Mauritania** | 0 (0-0) | 0.01 (0-0.03) | 0 (0-0) | 0 (0-0.01) | -3.72 (-4.71--2.73) |
| **Mauritius** | 0 (0-0) | 0 (0-0) | 0 (0-0) | 0 (0-0) | -1.65 (-5.29-2.14) |
| **Mexico** | 1 (1-1) | 0 (0-0) | 5 (2-6) | 0 (0-0.01) | 4.46 (3.7-5.24) |
| **Micronesia (Federated States of)** | 0 (0-0) | 0 (0-0) | 0 (0-0) | 0 (0-0) | -8.2 (-9.78--6.59) |
| **Monaco** | 0 (0-0) | 0.01 (0-0.03) | 0 (0-0) | 0.01 (0-0.01) | -3.22 (-3.59--2.86) |
| **Mongolia** | 1 (0-3) | 0.1 (0.02-0.22) | 1 (0-2) | 0.04 (0.01-0.08) | -4.02 (-4.4--3.64) |
| **Montenegro** | 0 (0-1) | 0.07 (0.04-0.11) | 0 (0-1) | 0.05 (0.02-0.08) | -1.99 (-2.27--1.71) |
| **Morocco** | 5 (0-16) | 0.03 (0-0.11) | 5 (-1-22) | 0.02 (0-0.07) | -1.96 (-2.4--1.51) |
| **Mozambique** | 0 (0-1) | 0 (0-0.01) | 0 (0-1) | 0 (0-0.01) | -1.9 (-2.27--1.53) |
| **Myanmar** | 3 (0-7) | 0.01 (0-0.03) | 2 (1-5) | 0.01 (0-0.01) | -2.37 (-3.02--1.72) |
| **Namibia** | 0 (0-0) | 0.01 (0-0.02) | 0 (0-0) | 0.01 (0-0.01) | -2.75 (-3.41--2.09) |
| **Nauru** | 0 (0-0) | 0 (0-0) | 0 (0-0) | 0 (0-0) | -4.79 (-6.4--3.15) |
| **Nepal** | 3 (1-5) | 0.02 (0.01-0.04) | 4 (2-6) | 0.02 (0.01-0.03) | -1.1 (-1.45--0.75) |
| **Netherlands** | 1 (1-1) | 0.01 (0-0.01) | 4 (3-5) | 0.01 (0.01-0.01) | 2.25 (0.22-4.33) |
| **New Zealand** | 1 (0-1) | 0.03 (0.01-0.04) | 1 (0-2) | 0.02 (0.01-0.03) | -1.32 (-2.19--0.44) |
| **Nicaragua** | 0 (0-0) | 0 (0-0) | 0 (0-0) | 0 (0-0) | -1.79 (-3.13--0.43) |
| **Niger** | 1 (0-2) | 0.01 (0-0.04) | 1 (0-2) | 0 (0-0.01) | -4.74 (-5.56--3.92) |
| **Nigeria** | 4 (1-11) | 0.01 (0-0.01) | 3 (1-7) | 0 (0-0) | -5.83 (-6.57--5.09) |
| **Niue** | 0 (0-0) | 0 (0-0) | 0 (0-0) | 0 (0-0) | -3.05 (-4.28--1.81) |
| **North Macedonia** | 1 (0-1) | 0.04 (0.02-0.07) | 1 (0-2) | 0.04 (0.02-0.07) | -1.75 (-2.21--1.29) |
| **Northern Mariana Islands** | 0 (0-0) | 0 (0-0) | 0 (0-0) | 0 (0-0) | 0.44 (-0.55-1.43) |
| **Norway** | 0 (0-1) | 0.01 (0.01-0.01) | 1 (0-1) | 0.01 (0-0.01) | 0.19 (-0.95-1.34) |
| **Oman** | 1 (0-1) | 0.06 (0.01-0.16) | 1 (0-1) | 0.03 (0-0.07) | -2.22 (-2.73--1.71) |
| **Pakistan** | 33 (10-69) | 0.04 (0.01-0.08) | 54 (16-108) | 0.03 (0.01-0.07) | -0.09 (-0.42-0.24) |
| **Palau** | 0 (0-0) | 0 (0-0) | 0 (0-0) | 0 (0-0) | -4.71 (-5.78--3.63) |
| **Palestine** | 0 (0-0) | 0.02 (0-0.03) | 0 (0-0) | 0.01 (0-0.02) | -2.31 (-2.79--1.82) |
| **Panama** | 0 (0-0) | 0 (0-0) | 0 (0-0) | 0 (0-0) | -0.89 (-2.03-0.27) |
| **Papua New Guinea** | 0 (0-1) | 0.01 (0-0.01) | 0 (0-1) | 0 (0-0.01) | -0.97 (-1.28--0.66) |
| **Paraguay** | 0 (0-0) | 0.01 (0-0.01) | 0 (0-0) | 0 (0-0.01) | -2.23 (-2.84--1.62) |
| **Peru** | 2 (0-3) | 0.01 (0-0.02) | 1 (0-1) | 0 (0-0) | -5.84 (-6.18--5.5) |
| **Philippines** | 0 (0-1) | 0 (0-0) | 0 (0-1) | 0 (0-0) | -2.38 (-3.4--1.36) |
| **Poland** | 15 (13-18) | 0.04 (0.04-0.05) | 20 (16-24) | 0.03 (0.02-0.04) | -1.9 (-2.55--1.24) |
| **Portugal** | 0 (0-0) | 0 (0-0) | 1 (0-2) | 0 (0-0.01) | 3.06 (0.76-5.41) |
| **Puerto Rico** | 0 (0-0) | 0 (0-0) | 0 (0-0) | 0 (0-0) | -1.36 (-3.14-0.45) |
| **Qatar** | 0 (0-0) | 0.05 (0-0.11) | 0 (0-0) | 0.02 (0-0.04) | -4.26 (-4.73--3.79) |
| **Republic of Korea** | 11 (7-16) | 0.04 (0.02-0.06) | 7 (4-10) | 0.01 (0.01-0.01) | -4.16 (-4.58--3.74) |
| **Republic of Moldova** | 0 (0-1) | 0.01 (0.01-0.02) | 1 (0-1) | 0.01 (0.01-0.01) | -1.88 (-2.43--1.32) |
| **Romania** | 62 (43-90) | 0.3 (0.22-0.42) | 168 (119-219) | 0.48 (0.34-0.63) | 0.19 (-0.38-0.76) |
| **Russian Federation** | 41 (25-51) | 0.03 (0.02-0.04) | 53 (39-69) | 0.03 (0.02-0.03) | -1.14 (-1.83--0.45) |
| **Rwanda** | 1 (0-1) | 0.01 (-0.01-0.02) | 0 (0-0) | 0 (0-0) | -6.12 (-6.79--5.45) |
| **Saint Kitts and Nevis** | 0 (0-0) | 0 (0-0) | 0 (0-0) | 0 (0-0) | 0.44 (-0.84-1.74) |
| **Saint Lucia** | 0 (0-0) | 0 (0-0.01) | 0 (0-0) | 0 (0-0) | -2.71 (-4.78--0.6) |
| **Saint Vincent and the Grenadines** | 0 (0-0) | 0 (0-0) | 0 (0-0) | 0 (0-0) | -1.63 (-3.51-0.28) |
| **Samoa** | 0 (0-0) | 0 (0-0) | 0 (0-0) | 0 (0-0) | -2.53 (-3.51--1.53) |
| **San Marino** | 0 (0-0) | 0.02 (0.01-0.04) | 0 (0-0) | 0.02 (0.01-0.03) | 0.57 (0.06-1.08) |
| **Sao Tome and Principe** | 0 (0-0) | 0 (0-0.01) | 0 (0-0) | 0 (0-0) | -4.11 (-5.23--2.98) |
| **Saudi Arabia** | 6 (1-12) | 0.08 (0.02-0.17) | 4 (1-9) | 0.02 (0-0.05) | -4.54 (-4.9--4.19) |
| **Senegal** | 0 (0-1) | 0.01 (0-0.01) | 0 (0-0) | 0 (0-0) | -3.29 (-4.28--2.28) |
| **Serbia** | 4 (2-7) | 0.05 (0.03-0.09) | 3 (2-5) | 0.02 (0.01-0.03) | -4.16 (-4.57--3.75) |
| **Seychelles** | 0 (0-0) | 0.01 (0.01-0.02) | 0 (0-0) | 0.01 (0-0.01) | -2.78 (-3.48--2.08) |
| **Sierra Leone** | 0 (0-0) | 0 (0-0.01) | 0 (0-0) | 0 (0-0) | -4.97 (-5.55--4.38) |
| **Singapore** | 0 (0-1) | 0.01 (0-0.02) | 0 (0-0) | 0 (0-0.01) | -3.47 (-4.73--2.19) |
| **Slovakia** | 1 (1-2) | 0.02 (0.01-0.03) | 2 (1-2) | 0.02 (0.01-0.03) | -0.77 (-0.95--0.59) |
| **Slovenia** | 0 (0-0) | 0.01 (0.01-0.02) | 1 (0-1) | 0.01 (0.01-0.02) | -1.46 (-2--0.9) |
| **Solomon Islands** | 0 (0-0) | 0 (0-0) | 0 (0-0) | 0 (0-0) | -5.11 (-6.11--4.09) |
| **Somalia** | 0 (0-1) | 0 (0-0.01) | 0 (0-0) | 0 (0-0) | -2.56 (-3.16--1.95) |
| **South Africa** | 4 (2-6) | 0.01 (0.01-0.02) | 3 (1-4) | 0.01 (0-0.01) | -3.86 (-4.29--3.42) |
| **South Sudan** | 0 (0-1) | 0 (0-0.01) | 0 (0-0) | 0 (0-0) | -5.74 (-6.77--4.7) |
| **Spain** | 1 (1-2) | 0 (0-0) | 16 (9-21) | 0.01 (0.01-0.02) | 4.36 (2.66-6.08) |
| **Sri Lanka** | 2 (1-5) | 0.03 (0.01-0.05) | 1 (0-2) | 0 (0-0.01) | -8.27 (-9.56--6.96) |
| **Sudan** | 3 (0-12) | 0.02 (0-0.09) | 2 (0-10) | 0.01 (0-0.05) | -3.06 (-3.81--2.29) |
| **Suriname** | 0 (0-0) | 0 (0-0) | 0 (0-0) | 0 (0-0) | -1.27 (-2.71-0.2) |
| **Sweden** | 6 (4-8) | 0.05 (0.04-0.07) | 5 (4-6) | 0.03 (0.02-0.04) | -2.24 (-2.83--1.64) |
| **Switzerland** | 0 (0-0) | 0 (0-0) | 1 (1-1) | 0.01 (0-0.01) | 1.33 (-0.45-3.15) |
| **Syrian Arab Republic** | 3 (0-9) | 0.04 (0-0.13) | 2 (0-8) | 0.02 (0-0.07) | -2.84 (-3.23--2.46) |
| **Taiwan (Province of China)** | 1 (0-2) | 0.01 (0-0.01) | 6 (2-10) | 0.02 (0.01-0.03) | 4.32 (3.15-5.5) |
| **Tajikistan** | 0 (0-0) | 0 (0-0) | 0 (0-0) | 0 (0-0) | -2.07 (-2.54--1.6) |
| **Thailand** | 1 (0-2) | 0 (0-0) | 3 (0-6) | 0 (0-0.01) | -1.01 (-2.26-0.25) |
| **Timor-Leste** | 0 (0-0) | 0.01 (-0.01-0.01) | 0 (0-0) | 0 (0-0.01) | -1.26 (-2.6-0.09) |
| **Togo** | 0 (0-0) | 0 (0-0.01) | 0 (0-0) | 0 (0-0) | -5.63 (-6.3--4.96) |
| **Tokelau** | 0 (0-0) | 0 (0-0) | 0 (0-0) | 0 (0-0) | -2.79 (-4.37--1.18) |
| **Tonga** | 0 (0-0) | 0 (0-0) | 0 (0-0) | 0 (0-0) | -4.1 (-5.67--2.51) |
| **Trinidad and Tobago** | 0 (0-0) | 0 (0-0.01) | 0 (0-0) | 0 (0-0) | -4.26 (-5.94--2.55) |
| **Tunisia** | 1 (0-5) | 0.03 (0-0.09) | 2 (0-8) | 0.02 (0-0.07) | -1.67 (-2.04--1.3) |
| **Turkmenistan** | 2 (1-3) | 0.08 (0.06-0.11) | 4 (3-6) | 0.09 (0.06-0.13) | 0.4 (-0.87-1.69) |
| **Tuvalu** | 0 (0-0) | 0 (0-0) | 0 (0-0) | 0 (0-0) | -5.22 (-6.25--4.17) |
| **Turkiye** | 4 (1-7) | 0.01 (0-0.02) | 5 (3-7) | 0.01 (0-0.01) | -1.49 (-1.95--1.02) |
| **Uganda** | 1 (0-2) | 0 (0-0.01) | 0 (0-1) | 0 (0-0) | -5 (-5.66--4.34) |
| **Ukraine** | 18 (13-23) | 0.03 (0.03-0.05) | 37 (26-51) | 0.05 (0.04-0.07) | 1.03 (0.81-1.25) |
| **United Arab Emirates** | 0 (0-1) | 0.08 (0.01-0.2) | 1 (0-2) | 0.03 (0-0.08) | 0.1 (-1.06-1.28) |
| **United Kingdom** | 30 (23-36) | 0.04 (0.03-0.05) | 30 (21-36) | 0.03 (0.02-0.03) | -2.13 (-2.58--1.68) |
| **United Republic of Tanzania** | 1 (-1-2) | 0 (-0.01-0.01) | 1 (0-2) | 0 (0-0) | -3.79 (-4.26--3.31) |
| **United States of America** | 54 (38-65) | 0.02 (0.01-0.02) | 51 (38-61) | 0.01 (0.01-0.02) | -1.51 (-1.98--1.04) |
| **United States Virgin Islands** | 0 (0-0) | 0.01 (0-0.01) | 0 (0-0) | 0 (0-0.01) | -1 (-2.26-0.27) |
| **Uruguay** | 0 (0-1) | 0.01 (0-0.02) | 0 (0-0) | 0.01 (0-0.01) | -2.9 (-3.56--2.23) |
| **Uzbekistan** | 4 (3-6) | 0.03 (0.02-0.04) | 4 (3-6) | 0.02 (0.01-0.02) | -2.13 (-2.78--1.47) |
| **Vanuatu** | 0 (0-0) | 0 (0-0) | 0 (0-0) | 0 (0-0) | -2.01 (-3.05--0.97) |
| **Venezuela (Bolivarian Republic of)** | 0 (0-0) | 0 (0-0) | 0 (0-0) | 0 (0-0) | -4.05 (-5.5--2.58) |
| **Viet Nam** | 4 (1-9) | 0.01 (0-0.02) | 6 (2-12) | 0.01 (0-0.02) | -1.65 (-2.34--0.95) |
| **Yemen** | 1 (0-5) | 0.02 (0-0.07) | 1 (-1-6) | 0.01 (-0.01-0.04) | -2.73 (-3.22--2.24) |
| **Zambia** | 0 (0-1) | 0 (-0.01-0.01) | 1 (0-2) | 0.01 (0-0.02) | 0.83 (0.31-1.36) |
| **Zimbabwe** | 0 (0-1) | 0.01 (-0.01-0.02) | 1 (0-2) | 0.01 (0-0.02) | 0.46 (0.01-0.91) |

No: number; ASMR: age-standardized deaths rate; EAPC: Estimated annual percent change. 95%UI:95%uncertainty interval.

**Supplementary Table 2. DALYs cases and age-standardized rate of Low-temperature-related myocarditis and their EAPCs from 1990 to 2021 at the countries and territories levels**

|  | **No.1990(95%UI)** | **ASDR.1990(95%UI)** | **No.2021(95%UI)** | **ASDR.2021(95%UI)** | **EAPC(%)(95%UI)** |
| --- | --- | --- | --- | --- | --- |
| **GBD region** |  |  |  |  |  |
| **Advanced Health System** | 13962 (10521-16294) | 1.08 (0.82-1.25) | 14280 (10784-16852) | 0.75 (0.58-0.89) | -1.85 (-2.34--1.36) |
| **Africa** | 2414 (599-5167) | 0.34 (0.1-0.75) | 1567 (475-3859) | 0.13 (0.04-0.36) | -3.43 (-3.71--3.15) |
| **African Region** | 1907 (381-3895) | 0.32 (0.08-0.6) | 1244 (378-2732) | 0.12 (0.03-0.27) | -3.66 (-3.97--3.36) |
| **America** | 3887 (2822-4759) | 0.54 (0.39-0.66) | 3189 (2244-3985) | 0.32 (0.22-0.4) | -1.89 (-2.36--1.43) |
| **Andean Latin America** | 133 (62-233) | 0.37 (0.17-0.61) | 59 (28-86) | 0.09 (0.04-0.14) | -5.05 (-5.35--4.74) |
| **Asia** | 46713 (32213-67336) | 1.5 (1.04-2.16) | 35609 (24069-50465) | 0.82 (0.55-1.17) | -2.01 (-2.21--1.81) |
| **Australasia** | 226 (15-313) | 1.16 (0.08-1.6) | 130 (47-174) | 0.41 (0.15-0.56) | -3.72 (-4.37--3.07) |
| **Basic Health System** | 37460 (27261-52277) | 1.7 (1.24-2.35) | 24214 (13298-33349) | 0.79 (0.44-1.09) | -2.69 (-2.94--2.44) |
| **Caribbean** | 51 (23-108) | 0.14 (0.06-0.28) | 46 (24-81) | 0.1 (0.05-0.19) | -1.42 (-2.21--0.63) |
| **Central Africa** | 191 (-17-418) | 0.24 (-0.01-0.48) | 127 (8-286) | 0.08 (0-0.2) | -3.99 (-4.51--3.46) |
| **Central Asia** | 711 (547-906) | 1.16 (0.88-1.5) | 1754 (1312-2335) | 1.86 (1.4-2.46) | 1.91 (1.18-2.65) |
| **Central Europe** | 3132 (2470-3843) | 2.59 (2.06-3.09) | 5450 (3959-6973) | 3.14 (2.26-3.99) | -0.63 (-1.08--0.17) |
| **Central Latin America** | 144 (54-206) | 0.08 (0.03-0.11) | 247 (-26-385) | 0.11 (-0.01-0.17) | 1.2 (0.68-1.71) |
| **Central Sub-Saharan Africa** | 152 (6-359) | 0.24 (0.01-0.49) | 107 (-4-224) | 0.09 (0-0.22) | -3.57 (-4.11--3.03) |
| **Commonwealth High Income** | 1367 (926-1601) | 1.25 (0.85-1.46) | 1111 (773-1309) | 0.69 (0.48-0.81) | -2.33 (-2.78--1.88) |
| **Commonwealth Low Income** | 860 (231-1707) | 0.42 (0.12-0.86) | 883 (342-1733) | 0.27 (0.1-0.55) | -1.09 (-1.52--0.66) |
| **Commonwealth Middle Income** | 8414 (2227-17610) | 0.7 (0.21-1.45) | 10210 (3115-19678) | 0.53 (0.16-1.02) | -0.73 (-1.05--0.42) |
| **East Asia** | 33125 (23543-46496) | 3.07 (2.18-4.31) | 20060 (10367-27972) | 1.43 (0.77-1.96) | -2.86 (-3.17--2.54) |
| **East Asia & Pacific - WB** | 35095 (25117-49199) | 2.03 (1.46-2.83) | 21614 (11302-29587) | 0.89 (0.48-1.21) | -2.97 (-3.23--2.71) |
| **Eastern Africa** | 732 (-45-1613) | 0.33 (-0.01-0.71) | 401 (58-945) | 0.11 (0.02-0.3) | -4.18 (-4.55--3.81) |
| **Eastern Europe** | 1447 (995-1747) | 0.69 (0.48-0.82) | 2120 (1597-2690) | 0.81 (0.6-1.03) | 0.05 (-0.41-0.52) |
| **Eastern Mediterranean Region** | 5268 (1709-10776) | 1.38 (0.45-2.8) | 5380 (1922-10039) | 0.82 (0.31-1.58) | -1.42 (-1.68--1.15) |
| **Eastern Sub-Saharan Africa** | 714 (-309-1715) | 0.27 (-0.11-0.61) | 401 (-73-840) | 0.1 (-0.01-0.22) | -4.02 (-4.34--3.69) |
| **Europe** | 9477 (7042-11167) | 1.15 (0.87-1.35) | 10832 (7949-13292) | 0.91 (0.67-1.1) | -1.73 (-2.29--1.16) |
| **Europe & Central Asia - WB** | 9813 (7355-11575) | 1.13 (0.86-1.33) | 12180 (9083-14644) | 0.97 (0.73-1.17) | -1.28 (-1.84--0.73) |
| **European Region** | 9842 (7372-11616) | 1.13 (0.86-1.32) | 12215 (9089-14680) | 0.97 (0.72-1.16) | -1.3 (-1.85--0.74) |
| **High-income Asia Pacific** | 1179 (827-1472) | 0.79 (0.55-0.99) | 913 (663-1061) | 0.48 (0.35-0.56) | -2.15 (-2.65--1.66) |
| **High-income North America** | 2980 (2137-3591) | 1.12 (0.8-1.35) | 2410 (1817-2877) | 0.73 (0.55-0.88) | -1.56 (-2.06--1.05) |
| **Latin America & Caribbean - WB** | 908 (600-1248) | 0.21 (0.14-0.29) | 780 (335-1118) | 0.12 (0.05-0.18) | -2.11 (-2.58--1.63) |
| **Limited Health System** | 10490 (3356-21344) | 0.66 (0.23-1.29) | 12174 (4232-22815) | 0.47 (0.16-0.87) | -0.92 (-1.21--0.63) |
| **Middle East & North Africa - WB** | 3104 (1136-5956) | 1.29 (0.46-2.47) | 2086 (846-3971) | 0.54 (0.22-1.08) | -2.62 (-2.94--2.3) |
| **Minimal Health System** | 579 (144-1399) | 0.4 (0.09-1.03) | 529 (113-1328) | 0.18 (0.04-0.5) | -2.95 (-3.23--2.68) |
| **North Africa and Middle East** | 3611 (1313-7566) | 1.13 (0.38-2.33) | 2579 (1113-5427) | 0.5 (0.22-1.05) | -2.51 (-2.79--2.22) |
| **North America** | 2980 (2136-3591) | 1.12 (0.8-1.35) | 2410 (1817-2877) | 0.73 (0.55-0.88) | -1.56 (-2.06--1.05) |
| **Northern Africa** | 562 (84-1972) | 0.51 (0.07-1.71) | 370 (6-1423) | 0.23 (0-0.92) | -2.53 (-2.84--2.22) |
| **Oceania** | 24 (4-46) | 0.28 (0.04-0.54) | 37 (-7-77) | 0.22 (-0.04-0.46) | -0.7 (-1.04--0.36) |
| **Region of the Americas** | 3887 (2822-4759) | 0.54 (0.39-0.66) | 3189 (2244-3985) | 0.32 (0.22-0.4) | -1.89 (-2.36--1.43) |
| **South-East Asia Region** | 7275 (2134-14901) | 0.59 (0.19-1.17) | 8257 (2864-15263) | 0.45 (0.16-0.81) | -0.77 (-1.08--0.45) |
| **South Asia** | 8420 (2189-17788) | 0.77 (0.21-1.62) | 10601 (3223-20478) | 0.64 (0.19-1.22) | -0.31 (-0.64-0.02) |
| **South Asia - WB** | 8709 (2353-18561) | 0.78 (0.21-1.66) | 10905 (3376-21091) | 0.64 (0.2-1.23) | -0.41 (-0.73--0.08) |
| **Southeast Asia** | 598 (195-1285) | 0.15 (0.05-0.31) | 467 (149-934) | 0.08 (0.03-0.15) | -3.04 (-3.57--2.5) |
| **Southern Africa** | 382 (58-662) | 0.39 (0.09-0.64) | 324 (95-602) | 0.19 (0.06-0.37) | -2.85 (-3.17--2.53) |
| **Southern Latin America** | 306 (176-409) | 0.62 (0.36-0.83) | 122 (76-150) | 0.18 (0.11-0.23) | -4.05 (-4.6--3.5) |
| **Southern Sub-Saharan Africa** | 253 (112-375) | 0.5 (0.24-0.76) | 201 (80-354) | 0.26 (0.11-0.46) | -2.66 (-3.01--2.31) |
| **Sub-Saharan Africa - WB** | 1859 (355-3911) | 0.3 (0.08-0.58) | 1199 (400-2645) | 0.1 (0.03-0.25) | -4.06 (-4.43--3.69) |
| **Tropical Latin America** | 277 (139-432) | 0.19 (0.1-0.3) | 307 (112-479) | 0.14 (0.05-0.22) | -2.05 (-2.91--1.19) |
| **Western Africa** | 547 (108-1400) | 0.24 (0.05-0.56) | 344 (69-854) | 0.06 (0.01-0.15) | -4.84 (-5.51--4.16) |
| **Western Europe** | 4467 (3105-5381) | 0.99 (0.7-1.17) | 2881 (2038-3490) | 0.46 (0.33-0.55) | -3.2 (-4.2--2.19) |
| **Western Pacific Region** | 34271 (24470-47974) | 2.41 (1.74-3.37) | 20781 (10878-28847) | 1.06 (0.58-1.44) | -3.05 (-3.33--2.77) |
| **Western Sub-Saharan Africa** | 605 (124-1536) | 0.24 (0.05-0.55) | 394 (72-987) | 0.06 (0.01-0.16) | -4.76 (-5.42--4.1) |
| **World Bank High Income** | 11971 (8939-14042) | 1.2 (0.91-1.4) | 11579 (8725-13645) | 0.77 (0.59-0.9) | -2.08 (-2.59--1.58) |
| **World Bank Low Income** | 1924 (745-3966) | 0.57 (0.24-1.16) | 1483 (637-3284) | 0.3 (0.14-0.67) | -2.41 (-2.59--2.22) |
| **World Bank Lower Middle Income** | 11385 (3883-22614) | 0.62 (0.23-1.16) | 13146 (5144-23736) | 0.45 (0.18-0.8) | -0.89 (-1.15--0.63) |
| **World Bank Upper Middle Income** | 37212 (26729-51378) | 1.94 (1.4-2.66) | 24988 (14048-34158) | 0.94 (0.54-1.29) | -2.64 (-2.9--2.39) |
| **Country** |  |  |  |  |  |
| **Afghanistan** | 191 (24-670) | 1.89 (0.23-6.53) | 280 (39-934) | 1.34 (0.17-4.45) | -1.06 (-1.26--0.87) |
| **Albania** | 63 (39-91) | 2.19 (1.32-3.26) | 48 (21-84) | 1.61 (0.72-2.66) | -1.9 (-2.25--1.54) |
| **Algeria** | 199 (32-676) | 0.95 (0.15-3.14) | 157 (17-616) | 0.43 (0.05-1.7) | -2.36 (-2.66--2.06) |
| **American Samoa** | 0 (0-0) | 0.01 (0-0.02) | 0 (0-0) | 0.01 (0.01-0.02) | -1.3 (-2.45--0.14) |
| **Andorra** | 0 (0-1) | 0.99 (0.54-1.71) | 0 (0-1) | 0.41 (0.21-0.7) | -3.04 (-3.2--2.87) |
| **Angola** | 35 (-16-100) | 0.3 (-0.14-0.7) | 35 (-10-76) | 0.11 (-0.03-0.25) | -3.24 (-3.85--2.63) |
| **Antigua and Barbuda** | 0 (0-0) | 0.24 (0.14-0.31) | 0 (0-0) | 0.1 (0.06-0.13) | -1.9 (-3.17--0.61) |
| **Argentina** | 230 (133-317) | 0.7 (0.41-0.97) | 87 (60-108) | 0.19 (0.13-0.24) | -4.3 (-4.83--3.78) |
| **Armenia** | 10 (7-14) | 0.33 (0.24-0.47) | 6 (3-9) | 0.16 (0.09-0.25) | -3.1 (-4.1--2.08) |
| **Australia** | 173 (-6-254) | 1.06 (-0.04-1.55) | 83 (24-116) | 0.31 (0.09-0.43) | -4.49 (-5.15--3.84) |
| **Austria** | 12 (10-14) | 0.14 (0.12-0.17) | 52 (42-61) | 0.43 (0.35-0.52) | 3.83 (1.67-6.05) |
| **Azerbaijan** | 169 (85-285) | 2.64 (1.3-4.5) | 261 (113-526) | 2.24 (1.03-4.34) | -1.77 (-2.19--1.35) |
| **Bahamas** | 0 (0-0) | 0.11 (0.05-0.16) | 0 (0-1) | 0.11 (0.07-0.15) | -1.28 (-2.75-0.2) |
| **Bahrain** | 1 (0-2) | 0.37 (0.1-0.73) | 1 (0-2) | 0.12 (0.04-0.23) | -3.94 (-4.37--3.51) |
| **Bangladesh** | 574 (129-1279) | 0.6 (0.13-1.34) | 732 (235-1501) | 0.48 (0.16-0.99) | -0.21 (-0.73-0.3) |
| **Barbados** | 0 (0-0) | 0.05 (0.03-0.07) | 0 (0-0) | 0.02 (0.01-0.03) | -1.76 (-3.6-0.12) |
| **Belarus** | 80 (41-128) | 0.74 (0.41-1.16) | 34 (23-45) | 0.29 (0.2-0.37) | -3.93 (-4.51--3.34) |
| **Belgium** | 16 (13-19) | 0.16 (0.12-0.18) | 55 (40-68) | 0.3 (0.21-0.37) | 2.11 (0.05-4.21) |
| **Belize** | 0 (0-1) | 0.18 (0.08-0.3) | 0 (0-0) | 0.04 (0.02-0.06) | -4.61 (-5.57--3.63) |
| **Benin** | 8 (0-26) | 0.11 (0-0.39) | 3 (0-8) | 0.01 (0-0.04) | -6.04 (-6.74--5.32) |
| **Bermuda** | 0 (0-0) | 0.15 (0.01-0.24) | 0 (0-0) | 0.13 (0.06-0.2) | -1.06 (-1.9--0.2) |
| **Bhutan** | 6 (3-10) | 1.02 (0.44-1.79) | 5 (2-9) | 0.74 (0.34-1.32) | -1.42 (-1.73--1.1) |
| **Bolivia (Plurinational State of)** | 35 (16-79) | 0.55 (0.27-1.09) | 23 (12-38) | 0.22 (0.11-0.37) | -3.15 (-3.32--2.98) |
| **Bosnia and Herzegovina** | 99 (52-176) | 2.42 (1.25-4.29) | 79 (35-150) | 1.75 (0.77-3.27) | -2.04 (-2.34--1.73) |
| **Botswana** | 6 (2-11) | 0.46 (0.14-0.91) | 6 (2-14) | 0.27 (0.09-0.59) | -2.51 (-3.01--2.01) |
| **Brazil** | 265 (134-416) | 0.19 (0.1-0.3) | 300 (108-466) | 0.14 (0.05-0.22) | -2.03 (-2.9--1.15) |
| **Brunei Darussalam** | 0 (0-1) | 0.15 (0.07-0.25) | 0 (0-0) | 0.06 (0.03-0.1) | -4.53 (-5.64--3.4) |
| **Bulgaria** | 167 (116-211) | 1.92 (1.34-2.42) | 312 (190-442) | 3.21 (1.97-4.56) | -0.34 (-1.13-0.45) |
| **Burkina Faso** | 29 (-3-100) | 0.24 (-0.03-0.75) | 9 (-4-33) | 0.03 (-0.02-0.12) | -5.3 (-6.36--4.24) |
| **Burundi** | 25 (-42-68) | 0.31 (-0.55-0.77) | 10 (-20-28) | 0.07 (-0.15-0.21) | -5.02 (-5.72--4.31) |
| **Cabo Verde** | 0 (-1-1) | 0.07 (-0.15-0.23) | 0 (0-1) | 0.04 (-0.06-0.13) | -2.5 (-3.94--1.04) |
| **Cambodia** | 9 (0-40) | 0.1 (0-0.37) | 10 (-4-33) | 0.07 (-0.03-0.23) | -4.1 (-5.53--2.66) |
| **Cameroon** | 27 (-4-59) | 0.21 (-0.03-0.47) | 20 (-6-50) | 0.06 (-0.02-0.14) | -4.39 (-5.05--3.72) |
| **Canada** | 115 (90-134) | 0.48 (0.38-0.56) | 174 (144-199) | 0.5 (0.41-0.57) | 0.54 (-0.29-1.38) |
| **Central African Republic** | 5 (1-13) | 0.15 (0.02-0.39) | 3 (1-8) | 0.06 (0.01-0.15) | -3.69 (-4.42--2.96) |
| **Chad** | 22 (2-66) | 0.27 (0.02-0.72) | 25 (1-88) | 0.1 (0-0.34) | -4.3 (-5.16--3.43) |
| **Chile** | 58 (34-73) | 0.43 (0.25-0.54) | 27 (12-36) | 0.14 (0.06-0.19) | -2.98 (-3.77--2.19) |
| **China** | 32605 (23030-45944) | 3.13 (2.22-4.41) | 19460 (10006-27376) | 1.44 (0.77-1.99) | -2.91 (-3.24--2.59) |
| **Colombia** | 44 (-2-67) | 0.12 (0-0.18) | 59 (-41-104) | 0.13 (-0.09-0.24) | -0.69 (-1.4-0.03) |
| **Comoros** | 1 (0-3) | 0.21 (0.07-0.42) | 1 (0-1) | 0.08 (0.03-0.17) | -4.22 (-4.69--3.75) |
| **Congo** | 6 (2-11) | 0.23 (0.1-0.43) | 3 (1-6) | 0.05 (0.02-0.12) | -4.54 (-5.12--3.96) |
| **Cook Islands** | 0 (0-0) | 0 (0-0) | 0 (0-0) | 0 (0-0) | -6.82 (-10.51--2.97) |
| **Costa Rica** | 3 (-4-6) | 0.11 (-0.14-0.22) | 5 (-7-11) | 0.1 (-0.15-0.21) | -3.17 (-4.26--2.08) |
| **Croatia** | 120 (87-145) | 2.48 (1.79-2.98) | 243 (159-332) | 3.29 (2.12-4.53) | -1.78 (-2.96--0.59) |
| **Cuba** | 10 (6-13) | 0.1 (0.06-0.13) | 6 (4-9) | 0.06 (0.04-0.09) | -2 (-2.94--1.06) |
| **Cyprus** | 4 (1-7) | 0.53 (0.15-1.02) | 2 (0-4) | 0.11 (0-0.23) | -5.89 (-6.41--5.36) |
| **Czechia** | 61 (50-74) | 0.56 (0.45-0.67) | 108 (72-152) | 0.68 (0.45-0.97) | -0.3 (-0.64-0.04) |
| **Cote d'Ivoire** | 22 (1-53) | 0.14 (0.01-0.34) | 9 (2-21) | 0.03 (0.01-0.07) | -5.21 (-5.95--4.47) |
| **Democratic People's Republic of Korea** | 480 (324-730) | 2.53 (1.67-3.87) | 470 (296-789) | 1.95 (1.24-3.34) | -0.71 (-0.96--0.47) |
| **Democratic Republic of the Congo** | 103 (-4-230) | 0.23 (-0.01-0.46) | 65 (1-155) | 0.09 (0-0.24) | -3.67 (-4.23--3.1) |
| **Denmark** | 16 (14-19) | 0.31 (0.26-0.37) | 29 (24-34) | 0.35 (0.29-0.41) | 0.35 (-1.3-2.02) |
| **Djibouti** | 1 (0-4) | 0.29 (0.01-0.8) | 2 (0-5) | 0.13 (0-0.4) | -3.45 (-4.14--2.75) |
| **Dominica** | 0 (0-0) | 0.26 (0.15-0.41) | 0 (0-0) | 0.13 (0.07-0.19) | -2.19 (-3.47--0.89) |
| **Dominican Republic** | 6 (3-13) | 0.07 (0.03-0.14) | 4 (-1-8) | 0.04 (-0.01-0.07) | -1.65 (-2.36--0.94) |
| **Ecuador** | 28 (19-35) | 0.31 (0.21-0.39) | 13 (6-21) | 0.08 (0.04-0.12) | -6.39 (-7.11--5.67) |
| **Egypt** | 38 (2-189) | 0.05 (0-0.25) | 11 (1-48) | 0.01 (0-0.05) | -5.43 (-5.85--5) |
| **El Salvador** | 1 (0-2) | 0.01 (0-0.03) | 0 (0-0) | 0 (0-0.01) | -4.13 (-5.63--2.61) |
| **Equatorial Guinea** | 1 (0-3) | 0.26 (0.09-0.52) | 0 (0-1) | 0.03 (0.01-0.06) | -7.96 (-8.5--7.43) |
| **Eritrea** | 12 (0-32) | 0.28 (-0.01-0.73) | 7 (1-17) | 0.11 (0.01-0.26) | -3.96 (-4.55--3.36) |
| **Estonia** | 4 (1-6) | 0.23 (0.05-0.38) | 2 (1-3) | 0.09 (0.04-0.12) | -3.88 (-4.38--3.38) |
| **Eswatini** | 4 (2-7) | 0.53 (0.23-0.9) | 3 (1-6) | 0.28 (0.11-0.49) | -2.47 (-2.9--2.03) |
| **Ethiopia** | 264 (-23-683) | 0.39 (-0.03-0.92) | 113 (3-229) | 0.11 (0-0.24) | -4.95 (-5.44--4.47) |
| **Fiji** | 1 (0-2) | 0.16 (0.03-0.29) | 1 (-2-1) | 0.07 (-0.19-0.16) | -2.57 (-3.86--1.26) |
| **Finland** | 37 (16-52) | 0.7 (0.3-1) | 37 (14-52) | 0.49 (0.19-0.71) | -0.63 (-1.95-0.71) |
| **France** | 31 (23-37) | 0.05 (0.04-0.06) | 150 (107-184) | 0.15 (0.11-0.19) | 3.67 (1.57-5.8) |
| **Gabon** | 2 (1-4) | 0.23 (0.09-0.42) | 1 (0-1) | 0.04 (0.01-0.08) | -5.75 (-6.28--5.22) |
| **Gambia** | 2 (1-5) | 0.19 (0.05-0.43) | 2 (1-4) | 0.06 (0.02-0.15) | -2.99 (-3.79--2.18) |
| **Georgia** | 124 (76-167) | 2.16 (1.33-2.9) | 77 (40-116) | 1.58 (0.82-2.38) | -1.78 (-2.65--0.9) |
| **Germany** | 396 (286-514) | 0.45 (0.34-0.57) | 634 (521-763) | 0.58 (0.48-0.69) | 0.73 (-0.51-1.99) |
| **Ghana** | 38 (8-79) | 0.22 (0.04-0.49) | 10 (1-22) | 0.03 (0-0.06) | -6.76 (-7.52--5.99) |
| **Greece** | 5 (1-8) | 0.05 (0.01-0.07) | 18 (8-25) | 0.13 (0.06-0.18) | 3.19 (0.96-5.48) |
| **Greenland** | 0 (0-1) | 0.53 (-0.65-1.78) | 0 (0-1) | 0.45 (-0.07-1.15) | -0.91 (-1.32--0.48) |
| **Grenada** | 0 (0-0) | 0.09 (0.05-0.14) | 0 (0-0) | 0.03 (0.02-0.04) | -3.74 (-5.62--1.83) |
| **Guam** | 0 (0-0) | 0 (0-0.01) | 0 (0-0) | 0 (0-0.03) | -2.87 (-4.58--1.14) |
| **Guatemala** | 18 (-20-35) | 0.17 (-0.18-0.3) | 16 (-25-35) | 0.11 (-0.17-0.23) | -1.43 (-1.99--0.87) |
| **Guinea** | 19 (4-48) | 0.23 (0.05-0.52) | 11 (4-23) | 0.06 (0.02-0.13) | -3.97 (-4.51--3.43) |
| **Guinea-Bissau** | 1 (0-4) | 0.09 (-0.03-0.32) | 0 (0-1) | 0.01 (-0.01-0.05) | -3.24 (-4.67--1.79) |
| **Guyana** | 3 (1-5) | 0.36 (0.11-0.63) | 3 (1-5) | 0.35 (0.07-0.66) | -0.14 (-1.62-1.36) |
| **Haiti** | 24 (6-71) | 0.3 (0.09-0.79) | 27 (11-58) | 0.2 (0.08-0.42) | -2.33 (-4.11--0.51) |
| **Honduras** | 4 (1-9) | 0.09 (0.01-0.19) | 3 (-6-7) | 0.03 (-0.07-0.08) | -2.21 (-4.89-0.54) |
| **Hungary** | 96 (66-127) | 0.87 (0.62-1.13) | 89 (59-121) | 0.61 (0.41-0.84) | -1.97 (-2.32--1.61) |
| **Iceland** | 0 (0-1) | 0.15 (0.09-0.2) | 1 (1-2) | 0.31 (0.15-0.45) | 2.12 (0.4-3.86) |
| **India** | 5642 (1335-12170) | 0.67 (0.17-1.41) | 6642 (1973-13028) | 0.53 (0.16-1.03) | -0.66 (-1.01--0.31) |
| **Indonesia** | 135 (24-340) | 0.09 (0.02-0.19) | 79 (-135-192) | 0.04 (-0.06-0.08) | -3.48 (-4.66--2.28) |
| **Iran (Islamic Republic of)** | 463 (212-810) | 1.13 (0.54-1.86) | 316 (147-487) | 0.42 (0.2-0.65) | -2.3 (-2.91--1.68) |
| **Iraq** | 1436 (412-2613) | 7.37 (2.22-13.26) | 1006 (312-1858) | 3.1 (0.99-5.62) | -2.58 (-2.97--2.19) |
| **Ireland** | 11 (8-13) | 0.31 (0.24-0.37) | 49 (36-60) | 0.78 (0.57-0.96) | 3.54 (1.55-5.58) |
| **Israel** | 4 (0-7) | 0.09 (-0.01-0.15) | 8 (0-12) | 0.07 (0-0.12) | -0.07 (-2.19-2.1) |
| **Italy** | 2625 (1754-3271) | 3.68 (2.48-4.58) | 594 (312-771) | 0.34 (0.18-0.46) | -9.04 (-10.62--7.44) |
| **Jamaica** | 2 (1-2) | 0.08 (0.06-0.1) | 1 (1-2) | 0.04 (0.03-0.06) | -1.93 (-2.66--1.19) |
| **Japan** | 592 (420-688) | 0.54 (0.38-0.63) | 684 (483-809) | 0.5 (0.35-0.58) | -1.22 (-2.06--0.37) |
| **Jordan** | 10 (3-18) | 0.28 (0.09-0.48) | 8 (0-14) | 0.08 (0-0.15) | -4.06 (-4.54--3.57) |
| **Kazakhstan** | 37 (24-56) | 0.24 (0.15-0.37) | 979 (687-1421) | 4.99 (3.49-7.16) | 12.55 (10.43-14.71) |
| **Kenya** | 31 (-39-65) | 0.12 (-0.13-0.25) | 20 (-16-59) | 0.05 (-0.04-0.14) | -3.01 (-3.48--2.54) |
| **Kiribati** | 0 (0-0) | 0.01 (0-0.02) | 0 (0-0) | 0.13 (0.02-0.21) | -0.07 (-4.7-4.79) |
| **Kuwait** | 19 (4-36) | 1.32 (0.31-2.48) | 23 (6-44) | 0.77 (0.18-1.48) | -2.02 (-2.75--1.29) |
| **Kyrgyzstan** | 41 (32-51) | 0.92 (0.7-1.15) | 68 (47-88) | 1.12 (0.77-1.48) | 1.09 (0.16-2.02) |
| **Lao People's Democratic Republic** | 15 (2-52) | 0.37 (0.04-1) | 18 (6-40) | 0.28 (0.09-0.61) | -1.86 (-2.58--1.14) |
| **Latvia** | 5 (3-7) | 0.19 (0.12-0.26) | 5 (3-7) | 0.18 (0.12-0.25) | -1.17 (-1.6--0.75) |
| **Lebanon** | 25 (9-48) | 0.97 (0.32-1.87) | 17 (4-29) | 0.29 (0.06-0.5) | -3.81 (-4.18--3.44) |
| **Lesotho** | 14 (7-22) | 0.95 (0.5-1.62) | 11 (6-18) | 0.63 (0.32-1.08) | -1.6 (-1.85--1.34) |
| **Liberia** | 7 (2-16) | 0.21 (0.08-0.44) | 3 (1-6) | 0.04 (0.01-0.1) | -4.79 (-5.43--4.15) |
| **Libya** | 48 (7-172) | 0.95 (0.13-3.45) | 21 (2-86) | 0.44 (0.03-1.77) | -2.38 (-2.82--1.93) |
| **Lithuania** | 7 (5-9) | 0.18 (0.12-0.25) | 6 (4-7) | 0.14 (0.1-0.18) | -1.49 (-2.07--0.91) |
| **Luxembourg** | 1 (1-1) | 0.22 (0.18-0.25) | 3 (2-3) | 0.32 (0.26-0.39) | 0.97 (-0.07-2.03) |
| **Madagascar** | 84 (-25-191) | 0.52 (-0.19-1.08) | 66 (-5-135) | 0.23 (-0.02-0.52) | -3.18 (-3.47--2.9) |
| **Malawi** | 34 (-32-96) | 0.22 (-0.25-0.56) | 23 (1-48) | 0.13 (0.01-0.29) | -2.74 (-3.2--2.28) |
| **Malaysia** | 10 (4-18) | 0.06 (0.02-0.11) | 6 (3-12) | 0.02 (0.01-0.05) | -5.08 (-5.98--4.16) |
| **Maldives** | 0 (0-0) | 0.02 (0.01-0.13) | 0 (0-0) | 0.01 (0-0.03) | -4.27 (-5.13--3.4) |
| **Mali** | 29 (0-81) | 0.26 (0-0.71) | 9 (-3-30) | 0.03 (-0.01-0.11) | -4.4 (-5.57--3.21) |
| **Malta** | 1 (-1-2) | 0.32 (-0.22-0.66) | 3 (-2-7) | 0.54 (-0.26-1.07) | 1.11 (-0.72-2.98) |
| **Marshall Islands** | 0 (0-0) | 0 (0-0.01) | 0 (0-0) | 0 (0-0) | -7.55 (-8.94--6.13) |
| **Mauritania** | 9 (2-22) | 0.36 (0.07-0.94) | 4 (1-11) | 0.08 (0.01-0.24) | -3.49 (-4.47--2.5) |
| **Mauritius** | 0 (0-0) | 0 (-0.02-0.01) | 0 (-1-1) | 0.02 (-0.1-0.06) | -0.19 (-3.87-3.63) |
| **Mexico** | 58 (39-75) | 0.06 (0.04-0.08) | 152 (50-217) | 0.13 (0.04-0.19) | 3.89 (3.1-4.69) |
| **Micronesia (Federated States of)** | 0 (0-0) | 0 (0-0.01) | 0 (0-0) | 0 (0-0) | -7.85 (-9.46--6.21) |
| **Monaco** | 0 (0-0) | 0.43 (0.1-0.7) | 0 (0-0) | 0.19 (0.09-0.31) | -3.41 (-3.75--3.06) |
| **Mongolia** | 45 (12-98) | 2.88 (0.73-6.74) | 34 (11-66) | 1.14 (0.38-2.21) | -3.95 (-4.33--3.57) |
| **Montenegro** | 12 (7-20) | 2.07 (1.14-3.32) | 9 (5-15) | 1.2 (0.62-2) | -2.56 (-2.82--2.3) |
| **Morocco** | 218 (20-820) | 0.91 (0.08-3.04) | 138 (-24-585) | 0.42 (-0.07-1.77) | -2.49 (-2.92--2.05) |
| **Mozambique** | 35 (6-89) | 0.19 (0.04-0.41) | 29 (10-56) | 0.1 (0.03-0.21) | -2.45 (-2.81--2.09) |
| **Myanmar** | 140 (26-410) | 0.38 (0.07-1) | 88 (21-191) | 0.18 (0.04-0.38) | -2.24 (-2.88--1.6) |
| **Namibia** | 4 (2-8) | 0.32 (0.13-0.61) | 5 (2-10) | 0.21 (0.07-0.42) | -2.52 (-3.18--1.86) |
| **Nauru** | 0 (0-0) | 0.01 (0-0.01) | 0 (0-0) | 0 (0-0) | -6.04 (-7.67--4.38) |
| **Nepal** | 171 (73-331) | 0.82 (0.36-1.48) | 152 (75-274) | 0.54 (0.26-0.96) | -1.16 (-1.5--0.82) |
| **Netherlands** | 24 (19-28) | 0.16 (0.13-0.19) | 64 (46-78) | 0.27 (0.19-0.32) | 1.27 (-0.79-3.37) |
| **New Zealand** | 53 (24-68) | 1.59 (0.74-2.08) | 47 (18-67) | 0.92 (0.35-1.31) | -1.89 (-2.69--1.07) |
| **Nicaragua** | 1 (0-3) | 0.03 (0.01-0.05) | 1 (0-1) | 0.01 (0-0.02) | -2.67 (-3.96--1.37) |
| **Niger** | 58 (4-186) | 0.53 (0.04-1.47) | 50 (3-147) | 0.16 (0.01-0.5) | -4.8 (-5.63--3.96) |
| **Nigeria** | 290 (58-802) | 0.25 (0.05-0.6) | 221 (48-519) | 0.07 (0.02-0.17) | -4.94 (-5.71--4.17) |
| **Niue** | 0 (0-0) | 0.1 (0.03-0.19) | 0 (0-0) | 0.11 (0-0.21) | -2.57 (-3.86--1.26) |
| **North Macedonia** | 24 (14-38) | 1.35 (0.8-2.1) | 25 (10-48) | 0.97 (0.43-1.87) | -2.52 (-2.95--2.09) |
| **Northern Mariana Islands** | 0 (0-0) | 0.04 (0.02-0.08) | 0 (0-0) | 0.04 (0.02-0.07) | 0.38 (-0.61-1.37) |
| **Norway** | 19 (10-25) | 0.46 (0.25-0.62) | 17 (11-22) | 0.26 (0.17-0.34) | -0.92 (-2-0.17) |
| **Oman** | 27 (3-72) | 1.75 (0.2-4.63) | 21 (1-54) | 0.73 (0.05-1.85) | -2.37 (-2.92--1.82) |
| **Pakistan** | 2028 (551-4411) | 1.58 (0.46-3.37) | 3071 (892-6034) | 1.34 (0.39-2.67) | 0.01 (-0.33-0.35) |
| **Palau** | 0 (0-0) | 0.01 (0-0.03) | 0 (0-0) | 0.01 (0-0.02) | -4.65 (-5.72--3.56) |
| **Palestine** | 9 (2-17) | 0.42 (0.1-0.77) | 8 (-1-14) | 0.2 (-0.01-0.39) | -2.39 (-2.86--1.92) |
| **Panama** | 0 (0-1) | 0.01 (0.01-0.03) | 1 (0-1) | 0.02 (0-0.03) | -1.03 (-2.16-0.11) |
| **Papua New Guinea** | 20 (2-41) | 0.37 (0.03-0.74) | 34 (-9-72) | 0.26 (-0.07-0.56) | -0.98 (-1.3--0.67) |
| **Paraguay** | 12 (5-21) | 0.25 (0.1-0.44) | 7 (2-12) | 0.1 (0.04-0.19) | -2.77 (-3.38--2.15) |
| **Peru** | 70 (16-132) | 0.35 (0.08-0.6) | 23 (5-39) | 0.07 (0.01-0.11) | -5.72 (-6.06--5.37) |
| **Philippines** | 21 (5-45) | 0.03 (0.01-0.07) | 20 (6-36) | 0.02 (0.01-0.04) | -2.44 (-3.43--1.45) |
| **Poland** | 605 (524-685) | 1.76 (1.52-1.99) | 427 (334-530) | 0.85 (0.67-1.06) | -2.98 (-3.38--2.59) |
| **Portugal** | 6 (1-9) | 0.07 (0.01-0.1) | 19 (6-28) | 0.12 (0.04-0.18) | 1.9 (-0.14-3.97) |
| **Puerto Rico** | 1 (0-1) | 0.01 (0.01-0.02) | 0 (0-1) | 0.01 (0.01-0.02) | -1.24 (-3.07-0.63) |
| **Qatar** | 3 (0-7) | 1.14 (0.08-2.54) | 5 (0-15) | 0.36 (0.02-0.94) | -3.97 (-4.36--3.58) |
| **Republic of Korea** | 577 (346-811) | 1.49 (0.92-2.11) | 219 (140-302) | 0.5 (0.31-0.67) | -3.58 (-3.85--3.31) |
| **Republic of Moldova** | 19 (16-23) | 0.46 (0.38-0.56) | 17 (12-22) | 0.35 (0.26-0.47) | -2.18 (-2.71--1.65) |
| **Romania** | 1677 (1212-2268) | 7.59 (5.66-9.92) | 3910 (2696-5205) | 13.17 (9.08-17.56) | 0.61 (0.02-1.2) |
| **Russian Federation** | 906 (567-1112) | 0.68 (0.42-0.84) | 1402 (973-1873) | 0.78 (0.54-1.06) | -0.01 (-0.63-0.61) |
| **Rwanda** | 39 (-34-101) | 0.39 (-0.36-0.94) | 9 (-14-22) | 0.07 (-0.11-0.17) | -6.75 (-7.45--6.06) |
| **Saint Kitts and Nevis** | 0 (0-0) | 0.04 (0.02-0.05) | 0 (0-0) | 0.03 (0.02-0.04) | -0.14 (-1.41-1.14) |
| **Saint Lucia** | 0 (0-0) | 0.17 (0.03-0.24) | 0 (0-0) | 0.06 (0.01-0.08) | -2.54 (-4.59--0.46) |
| **Saint Vincent and the Grenadines** | 0 (0-0) | 0.04 (0.01-0.06) | 0 (0-0) | 0.02 (0.01-0.03) | -2.44 (-4.29--0.56) |
| **Samoa** | 0 (0-0) | 0.11 (0.03-0.22) | 0 (0-0) | 0.08 (0.04-0.12) | -2.31 (-3.3--1.31) |
| **San Marino** | 0 (0-0) | 0.61 (0.32-1.08) | 0 (0-0) | 0.42 (0.22-0.66) | -0.2 (-0.62-0.21) |
| **Sao Tome and Principe** | 0 (0-0) | 0.14 (0.05-0.27) | 0 (0-0) | 0.02 (0.01-0.07) | -3.45 (-4.62--2.27) |
| **Saudi Arabia** | 308 (63-657) | 2.35 (0.46-4.82) | 157 (35-352) | 0.58 (0.12-1.32) | -5.01 (-5.38--4.64) |
| **Senegal** | 24 (2-61) | 0.24 (0.03-0.57) | 10 (1-27) | 0.06 (0.01-0.16) | -3.01 (-4.03--1.99) |
| **Serbia** | 114 (60-168) | 1.32 (0.71-1.95) | 71 (41-106) | 0.57 (0.33-0.88) | -3.72 (-4.06--3.38) |
| **Seychelles** | 0 (0-0) | 0.3 (0.17-0.45) | 0 (0-0) | 0.15 (0.09-0.23) | -2.82 (-3.5--2.13) |
| **Sierra Leone** | 14 (4-33) | 0.22 (0.06-0.44) | 7 (2-15) | 0.06 (0.02-0.13) | -4.7 (-5.28--4.11) |
| **Singapore** | 9 (3-30) | 0.29 (0.09-0.97) | 10 (1-13) | 0.15 (0.02-0.2) | -4.14 (-5.39--2.87) |
| **Slovakia** | 36 (24-52) | 0.69 (0.47-1) | 40 (24-59) | 0.56 (0.34-0.84) | -1.07 (-1.24--0.89) |
| **Slovenia** | 7 (6-9) | 0.32 (0.25-0.4) | 11 (7-15) | 0.28 (0.17-0.39) | -2.19 (-2.81--1.56) |
| **Solomon Islands** | 0 (0-0) | 0.04 (0.01-0.09) | 0 (0-0) | 0.01 (0-0.03) | -5.28 (-6.28--4.27) |
| **Somalia** | 14 (-1-47) | 0.13 (-0.01-0.39) | 13 (3-31) | 0.05 (0.01-0.13) | -2.97 (-3.58--2.35) |
| **South Africa** | 200 (93-304) | 0.55 (0.26-0.83) | 115 (52-194) | 0.21 (0.1-0.35) | -3.74 (-4.13--3.34) |
| **South Sudan** | 15 (0-59) | 0.18 (-0.01-0.64) | 7 (-1-24) | 0.06 (-0.01-0.17) | -5.71 (-6.77--4.64) |
| **Spain** | 35 (20-44) | 0.09 (0.05-0.12) | 199 (116-262) | 0.24 (0.14-0.31) | 3.16 (1.18-5.17) |
| **Sri Lanka** | 99 (30-188) | 0.71 (0.22-1.35) | 24 (4-51) | 0.11 (0.02-0.23) | -8.13 (-9.4--6.85) |
| **Sudan** | 136 (1-734) | 0.63 (0.01-3.06) | 98 (0-467) | 0.29 (0-1.38) | -3.3 (-4.04--2.54) |
| **Suriname** | 0 (0-0) | 0.04 (0.01-0.09) | 0 (0-0) | 0.02 (0-0.04) | -1.58 (-3.02--0.11) |
| **Sweden** | 200 (137-254) | 2.09 (1.46-2.66) | 134 (98-169) | 1.04 (0.76-1.31) | -2.45 (-3.06--1.85) |
| **Switzerland** | 10 (8-12) | 0.15 (0.12-0.18) | 19 (14-23) | 0.14 (0.11-0.17) | -0.55 (-2.17-1.1) |
| **Syrian Arab Republic** | 153 (26-485) | 1.19 (0.18-3.79) | 58 (0-242) | 0.47 (0-2) | -3.2 (-3.68--2.71) |
| **Taiwan (Province of China)** | 40 (18-63) | 0.24 (0.1-0.37) | 130 (50-204) | 0.48 (0.18-0.76) | 4.67 (3.46-5.88) |
| **Tajikistan** | 1 (1-2) | 0.02 (0.01-0.04) | 2 (1-3) | 0.02 (0.01-0.03) | -1.54 (-1.86--1.23) |
| **Thailand** | 28 (1-69) | 0.06 (0-0.14) | 65 (8-138) | 0.09 (0.01-0.2) | -0.52 (-1.78-0.76) |
| **Timor-Leste** | 1 (-2-4) | 0.14 (-0.24-0.39) | 1 (-1-2) | 0.07 (-0.07-0.19) | -1.29 (-2.63-0.07) |
| **Togo** | 5 (0-16) | 0.11 (0-0.36) | 2 (0-4) | 0.02 (0-0.05) | -5.48 (-6.15--4.81) |
| **Tokelau** | 0 (0-0) | 0.01 (0-0.05) | 0 (0-0) | 0.02 (0.01-0.09) | -2.58 (-4.27--0.85) |
| **Tonga** | 0 (0-0) | 0.02 (0-0.03) | 0 (0-0) | 0.01 (0-0.01) | -3.91 (-5.48--2.31) |
| **Trinidad and Tobago** | 2 (0-3) | 0.18 (0.04-0.24) | 1 (0-2) | 0.09 (0.01-0.14) | -4.21 (-5.86--2.52) |
| **Tunisia** | 51 (6-188) | 0.72 (0.08-2.75) | 40 (1-168) | 0.34 (0.01-1.43) | -2.62 (-2.98--2.27) |
| **Turkmenistan** | 87 (62-124) | 2.85 (2-4.11) | 163 (111-232) | 3.26 (2.23-4.65) | 0.56 (-0.69-1.82) |
| **Tuvalu** | 0 (0-0) | 0.02 (0.01-0.09) | 0 (0-0) | 0.01 (0-0.01) | -5.13 (-6.18--4.07) |
| **Turkiye** | 185 (79-367) | 0.33 (0.14-0.65) | 125 (64-191) | 0.16 (0.08-0.24) | -2.46 (-2.82--2.1) |
| **Uganda** | 61 (-33-174) | 0.22 (-0.15-0.55) | 22 (-14-49) | 0.04 (-0.03-0.1) | -5.34 (-5.98--4.7) |
| **Ukraine** | 426 (312-544) | 0.8 (0.6-1.02) | 654 (469-895) | 1.15 (0.83-1.56) | 0.75 (0.51-0.99) |
| **United Arab Emirates** | 27 (4-68) | 2.25 (0.32-5.44) | 32 (3-78) | 0.8 (0.07-1.97) | -1.31 (-2.1--0.51) |
| **United Kingdom** | 1009 (764-1187) | 1.76 (1.34-2.05) | 791 (552-964) | 1.01 (0.71-1.23) | -2.34 (-2.75--1.93) |
| **United Republic of Tanzania** | 72 (-88-186) | 0.2 (-0.31-0.47) | 41 (-13-89) | 0.07 (-0.02-0.16) | -4.02 (-4.49--3.54) |
| **United States of America** | 2865 (2041-3464) | 1.19 (0.85-1.44) | 2236 (1660-2695) | 0.75 (0.56-0.91) | -1.67 (-2.16--1.18) |
| **United States Virgin Islands** | 0 (0-1) | 0.28 (0.07-0.5) | 0 (0-0) | 0.16 (0.08-0.28) | -0.56 (-1.82-0.71) |
| **Uruguay** | 18 (4-28) | 0.6 (0.14-0.91) | 8 (1-12) | 0.2 (0.03-0.31) | -4.06 (-4.67--3.45) |
| **Uzbekistan** | 194 (137-256) | 1.04 (0.74-1.4) | 164 (113-219) | 0.51 (0.35-0.68) | -2.2 (-2.87--1.53) |
| **Vanuatu** | 0 (0-0) | 0.1 (0.03-0.19) | 0 (0-0) | 0.07 (0.02-0.13) | -2.1 (-3.13--1.06) |
| **Venezuela (Bolivarian Republic of)** | 14 (6-22) | 0.07 (0.03-0.11) | 11 (2-19) | 0.04 (0.01-0.08) | -4.22 (-5.69--2.71) |
| **Viet Nam** | 139 (48-294) | 0.25 (0.08-0.55) | 154 (52-322) | 0.18 (0.06-0.36) | -1.67 (-2.34--1) |
| **Yemen** | 62 (3-295) | 0.47 (0.03-1.86) | 55 (-31-264) | 0.23 (-0.13-1.06) | -2.94 (-3.43--2.45) |
| **Zambia** | 25 (-37-80) | 0.22 (-0.32-0.64) | 36 (-1-107) | 0.22 (0-0.73) | -0.08 (-0.55-0.38) |
| **Zimbabwe** | 26 (-21-56) | 0.28 (-0.24-0.65) | 61 (-11-126) | 0.41 (-0.07-0.89) | 1.21 (0.71-1.71) |

DALYs: disability-adjusted life years; No: number; ASDR: age-standardized DALYs rate; EAPC: Estimated annual percent change. 95%UI:95%uncertainty interval.

**Supplementary Table 3. Utilize the BAPC model to predict the number of deaths and DALYs, age-standardized rates (per 100 000 persons) of deaths and DALYs associated with low temperature related to myocarditis for different genders**

| **year** | **sex** | **ASMR** | **Numer of deaths cases** | **ASDR** | **Numer of DALYs cases** |
| --- | --- | --- | --- | --- | --- |
| **2022** | **Male** | **0.030285** | **1201.402** | **0.828613** | **32870.67422** |
| **2023** | **Male** | **0.030509** | **1222.089** | **0.818835** | **32799.42541** |
| **2024** | **Male** | **0.03074** | **1242.99** | **0.809324** | **32725.66486** |
| **2025** | **Male** | **0.030978** | **1264.152** | **0.800268** | **32656.92256** |
| **2026** | **Male** | **0.031219** | **1285.277** | **0.7912** | **32573.93041** |
| **2027** | **Male** | **0.031459** | **1306.284** | **0.782138** | **32477.37825** |
| **2028** | **Male** | **0.0317** | **1327.234** | **0.773166** | **32371.01474** |
| **2029** | **Male** | **0.031945** | **1348.201** | **0.764352** | **32258.33239** |
| **2030** | **Male** | **0.032196** | **1369.28** | **0.755877** | **32147.21883** |
| **2031** | **Male** | **0.032447** | **1390.239** | **0.747309** | **32019.68651** |
| **2032** | **Male** | **0.032697** | **1411.035** | **0.738679** | **31877.30998** |
| **2033** | **Male** | **0.032949** | **1431.741** | **0.730076** | **31723.9194** |
| **2034** | **Male** | **0.033205** | **1452.448** | **0.721569** | **31563.07398** |
| **2035** | **Male** | **0.033466** | **1473.26** | **0.713339** | **31403.23677** |
| **2036** | **Male** | **0.033727** | **1493.915** | **0.705009** | **31228.2049** |
| **2037** | **Male** | **0.033986** | **1514.354** | **0.696633** | **31040.52617** |
| **2038** | **Male** | **0.034246** | **1534.632** | **0.688292** | **30844.0734** |
| **2039** | **Male** | **0.034506** | **1554.788** | **0.680034** | **30641.23926** |
| **2040** | **Male** | **0.034768** | **1574.854** | **0.672012** | **30439.16138** |
| **2041** | **Male** | **0.035025** | **1594.496** | **0.663872** | **30222.08534** |
| **2042** | **Male** | **0.035275** | **1613.612** | **0.655663** | **29992.35453** |
| **2043** | **Male** | **0.035519** | **1632.226** | **0.647437** | **29752.44308** |
| **2044** | **Male** | **0.035756** | **1650.338** | **0.639201** | **29502.76469** |
| **2045** | **Male** | **0.035988** | **1667.959** | **0.631086** | **29249.35897** |
| **2046** | **Male** | **0.036208** | **1684.77** | **0.622786** | **28978.17501** |
| **2022** | **Female** | **0.021371** | **842.8273** | **0.550172** | **21697.80838** |
| **2023** | **Female** | **0.021521** | **857.1687** | **0.540188** | **21515.59355** |
| **2024** | **Female** | **0.021676** | **871.7169** | **0.530437** | **21331.67384** |
| **2025** | **Female** | **0.02184** | **886.5702** | **0.521029** | **21150.58018** |
| **2026** | **Female** | **0.022005** | **901.4293** | **0.511746** | **20963.47054** |
| **2027** | **Female** | **0.02217** | **916.2142** | **0.502615** | **20771.64004** |
| **2028** | **Female** | **0.022335** | **930.9531** | **0.49366** | **20576.43977** |
| **2029** | **Female** | **0.022502** | **945.6863** | **0.484906** | **20379.41301** |
| **2030** | **Female** | **0.022672** | **960.5006** | **0.476442** | **20184.5907** |
| **2031** | **Female** | **0.02284** | **975.1446** | **0.468053** | **19983.28777** |
| **2032** | **Female** | **0.023005** | **989.562** | **0.459757** | **19776.49461** |
| **2033** | **Female** | **0.023168** | **1003.808** | **0.451582** | **19565.5862** |
| **2034** | **Female** | **0.023331** | **1017.937** | **0.443552** | **19351.92082** |
| **2035** | **Female** | **0.023496** | **1032.031** | **0.435752** | **19139.58585** |
| **2036** | **Female** | **0.023658** | **1045.856** | **0.427993** | **18920.63504** |
| **2037** | **Female** | **0.023815** | **1059.374** | **0.420302** | **18696.52702** |
| **2038** | **Female** | **0.02397** | **1072.665** | **0.412712** | **18468.92792** |
| **2039** | **Female** | **0.024125** | **1085.787** | **0.405247** | **18239.14123** |
| **2040** | **Female** | **0.02428** | **1098.816** | **0.397997** | **18011.52379** |
| **2041** | **Female** | **0.024433** | **1111.535** | **0.390807** | **17779.2989** |
| **2042** | **Female** | **0.024581** | **1123.924** | **0.383714** | **17544.45547** |
| **2043** | **Female** | **0.024728** | **1136.051** | **0.376737** | **17307.98377** |
| **2044** | **Female** | **0.024874** | **1147.955** | **0.369878** | **17070.2306** |
| **2045** | **Female** | **0.02502** | **1159.693** | **0.363207** | **16834.60225** |
| **2046** | **Female** | **0.025163** | **1171.04** | **0.356572** | **16594.32239** |

Abbreviations: BAPC, Bayesian age-period-cohort analysis（BAPC）. DALYs：disability-adjusted life years ；ASMR： Age-standardized mortality rate；ASDR: age-standardized DALYs rate.
